# Supplementary material for: Penigrisacids A–D, Four New Sesquiterpenes from the Deep-Sea-Derived Penicillium griseofulvum
Source: Mar Drugs. 2019 Aug 29;17(9):507. doi: 10.3390/md17090507 (PMC6780263; doi:10.3390/md17090507)
Supplement: Supplementary file 1 [file marinedrugs-17-00507-s001.pdf]

Supplementary Materials

Penigrisacids A–D, Four New Sesquiterpenes from the Deep-Sea-Derived *Penicillium griseofulvum*

Cui-Ping Xing [1], Chun-Lan Xie [1], Jin-Mei Xia [1], Qing-Mei Liu [2], Wei-Xiang Lin [1], De-Zan Ye [1], Guang-Ming Liu [2], and Xian-Wen Yang [1,*]

[1] Key Laboratory of Marine Biogenetic Resources, South China Sea Bio-Resource Exploitation and Utilization Collaborative Innovation Center, Third Institute of Oceanography, Ministry of Natural Resources, 184 Daxue Road, Xiamen 361005, China; xingcuiping123@126.com (C.-P. X.); xiechunlanxx@163.com (C.-L. X.); xiajinmei@tio.org.cn (J.-M. X.); 15989178371@163.com (W.-X. L.); yedezan@tio.org.cn (D.-Z. Y.)

[2] College of Food and Biological Engineering, Jimei University, 43 Yindou Road, Xiamen 361021, China; liuqingmei1229@163.com (Q.-M. L.); gmliu@jmu.edu.cn (G.-M. L.)

* Correspondence: yangxianwen@tio.org.cn; Tel.: +86-592-2195319

Content

Table S1. Energy analysis for 2*S*,3*R*,6*S*,7*R*-**1**, (6*S*,7*R*,10*S*,11*S*)-**2**, and (2*R*,3*R*,6*S*,7*S*,10*R*)-**3**.

Table S2. Calculated ECD data for 2*S*,3*R*,6*S*,7*R*-**1** (**1a**) and (6*S*,7*R*,10*S*,11*S*)-**2** (**2a**)

Figure S1. B3LYP-SCRF (PCM, ACN)/6-31G(d) optimized lowest energy conformers for 2*S*,3*R*,6*S*,7*R*-**1** (**1a**), (6*S*,7*R*,10*S*,11*S*)-**2**, and (2*R*,3*R*,6*S*,7*S*,10*R*)-**3**.

Figure S2. The CD spectra of compounds **3**–**5** in ACN.

Figure S3. ^{1}H NMR spectrum of **1** in CD_3OD.

Figure S4. ^{13}C NMR spectrum of **1** in CD_3OD.

Figure S5. HSQC spectrum of **1** in CD_3OD.

Figure S6. COSY NMR spectrum of **1** in CD_3OD.

Figure S7. HMBC NMR spectrum of **1** in CD_3OD.

Figure S8. NOESY NMR spectrum of **1** in CD_3OD.

Figure S9. The HR-ESI-MS spectrum of **1**.

Figure S10. ^{1}H NMR spectrum of **2** in CD_3OD.

Figure S11. ^{13}C NMR spectrum of **2** in CD_3OD.

Figure S12. HSQC spectrum of **2** in CD_3OD.

Figure S13. COSY NMR spectrum of **2** in CD_3OD.

Figure S14. HMBC NMR spectrum of **2** in CD_3OD.

Figure S15. NOESY NMR spectrum of **2** in CD_3OD.

Figure S16. The HR-ESI-MS spectrum of **2**.

Figure S17. ^{1}H NMR spectrum of **3** in DMSO-d_6.

Figure S18. ^{13}C NMR spectrum of **3** in DMSO-d_6.

Figure S19. HSQC spectrum of **3** in DMSO-d_6.

Figure S20. COSY NMR spectrum of **3** in DMSO-d_6.

Figure S21. HMBC NMR spectrum of **3** in DMSO-d_6.

Figure S22. NOESY NMR spectrum of **3** in DMSO-d_6.

Figure S23. The HR-ESI-MS spectrum of **3**.

Figure S24. ^{1}H NMR spectrum of **4** in CD_3OD.

Figure S25. ^{13}C NMR spectrum of **4** in CD_3OD.

Figure S26. HSQC spectrum of **4** in CD_3OD.

Figure S27. COSY NMR spectrum of **4** in CD_3OD.

Figure S28. HMBC NMR spectrum of **4** in CD_3OD.

Figure S29. NOESY NMR spectrum of **4** in CD_3OD.

Figure S30. The HR-ESI-MS spectrum of **4**.

Table S1. Energy analysis for 2*S*,3*R*,6*S*,7*R*-**1**, (6*S*,7*R*,10*S*,11*S*)-**2**, and (2*R*,3*R*,6*S*,7*S*,10*R*)-**3**.

Compounds	conformers	Gibbs free energy (298.15 K)		
		G (Hartree)	ΔE (kcal/mol)	Population (%)
**1**	C1	-885.814127	0	100
**2**	C1	-887.0162631	0	59.96
	C2	-887.0152285	0.001035	20.02
	C3	-887.0152282	0.001035	20.02
**3**	C1	-962.2614876	0	52.37
	C2	-962.2613983	0.00008937	47.63

Table S2. Calculated ECD data for 2*S*,3*R*,6*S*,7*R*-**1** (**1a**) and (6*S*,7*R*,10*S*,11*S*)-**2** (**2a**)

State	Excitation energies (ev)				Rotatory strength [a]			
	**1**-C1	**2**-C1	**2**-C2	**2**-C3	**1**-C1	**2**-C1	**2**-C2	**2**-C3
1	5.3386	4.8494	4.8272	4.8275	5.9147	12.8201	-6.5465	-6.4019
2	5.6098	5.0891	5.0568	5.0574	1.5175	-39.5845	-7.6845	-7.7548
3	5.6571	5.4380	5.4254	5.4254	1.4859	24.4594	-2.1097	-2.1647
4	5.9040	5.7025	5.7052	5.7059	-26.2110	2.3496	1.8929	1.9463
5	6.0490	5.8931	5.8368	5.8367	-19.7277	16.4094	5.2373	5.2511
6	6.1331	6.0712	6.0482	6.0486	86.8292	-20.3495	-18.0219	-18.1252
7	6.1920	6.1616	6.1704	6.1707	-2.5575	-9.4667	-7.3380	-7.1236
8	6.2694	6.2215	6.1878	6.1883	5.3327	-4.6239	-0.6867	-0.7401
9	6.3591	6.3439	6.3660	6.3666	-3.5681	-2.7571	-3.6331	-3.6922
10	6.5186	6.4326	6.4180	6.4180	-13.3508	24.5131	20.3259	20.4596
11	6.5400	6.5040	6.5172	6.5183	-6.1392	-0.8312	0.4324	0.4102
12	6.6821	6.6334	6.6006	6.6007	-3.6383	29.6529	20.4106	20.3614
13	6.6953	6.6602	6.6420	6.6419	3.6735	-7.6371	-4.7818	-4.9282
14	6.7627	6.7227	6.7220	6.7223	-0.6138	-40.5224	-13.0492	-13.0714
15	6.7899	6.7369	6.7459	6.7463	-0.2693	22.7328	-1.8263	-2.0245
16	6.8946	6.7896	6.7941	6.7944	-4.9629	2.3344	0.2456	0.3429
17	6.9521	6.8813	6.8291	6.8295	-11.3712	-13.2261	-15.5354	-15.4001
18	6.9882	6.8967	6.8945	6.8947	7.8980	1.0085	6.3925	6.3062
19	7.0152	6.9080	6.9061	6.9061	2.7649	5.6400	-8.4112	-8.3278
20	7.0436	6.9896	6.9745	6.9746	-6.1447	4.1590	-6.0588	-5.8168
21	7.1016	7.0294	7.0454	7.0455	4.3433	-29.5758	44.2191	44.2126
22	7.1203	7.1363	7.1145	7.1144	-0.7839	-4.4138	-3.8099	-3.8801
23	7.1817	7.1959	7.1828	7.1829	7.0059	5.1211	5.9225	5.8723
24	7.2850	7.2038	7.1948	7.1948	11.4784	22.6396	0.6583	0.8543
25	7.3096	7.2167	7.2080	7.2082	-6.3801	3.1666	26.1300	25.9073
26	7.3168	7.2373	7.2280	7.2280	2.3336	0.5304	-5.2686	-5.2962
27	7.3593	7.3057	7.3086	7.3086	-0.4547	-12.4655	-8.8393	-8.6322
28	7.3708	7.3223	7.3278	7.3279	-10.0520	1.7436	-38.9753	-39.6880
29	7.4080	7.3693	7.3410	7.3412	-9.1630	-5.6420	4.3430	4.6839
30	7.4528	7.4067	7.3889	7.3891	2.6219	12.0279	-3.1905	-3.0816

[a] R(velocity) 10**-40 erg-esu-cm

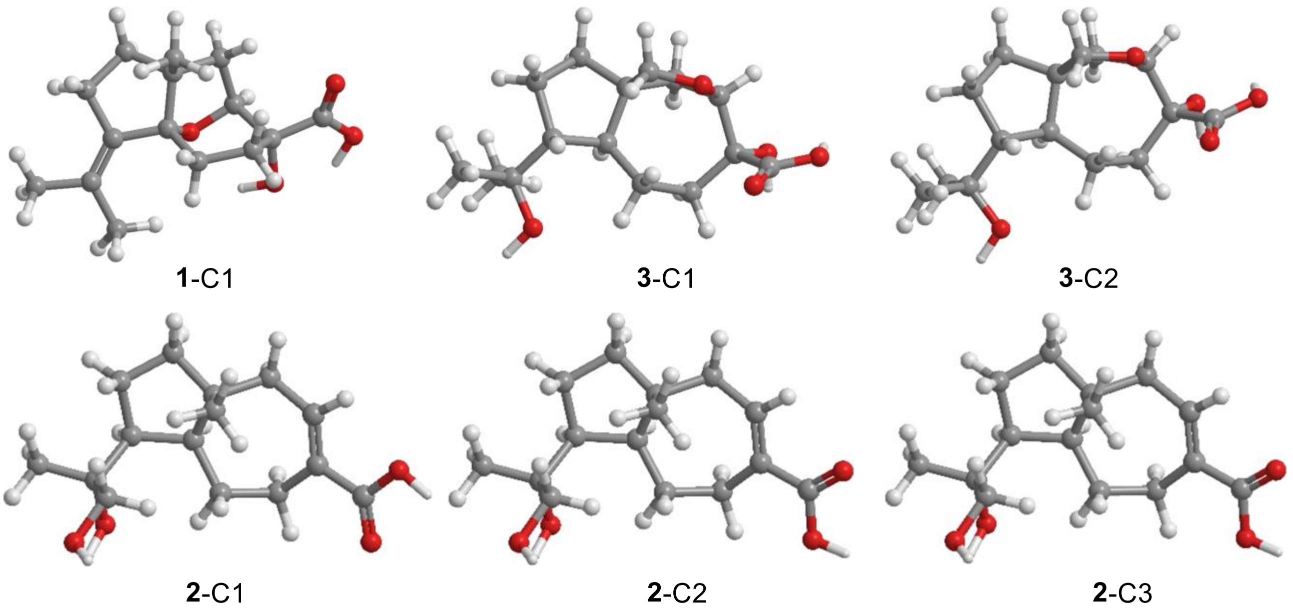

Figure S1. B3LYP-SCRF (PCM, ACN)/6-31G(d) optimized lowest energy conformers for 2*S*,3*R*,6*S*,7*R*-**1** (**1a**), (6*S*,7*R*,10*S*,11*S*)-**2**, and (2*R*,3*R*,6*S*,7*S*,10*R*)-**3**.

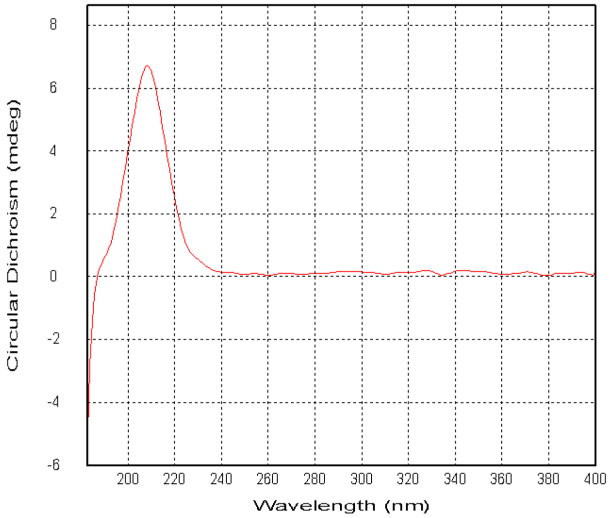

3

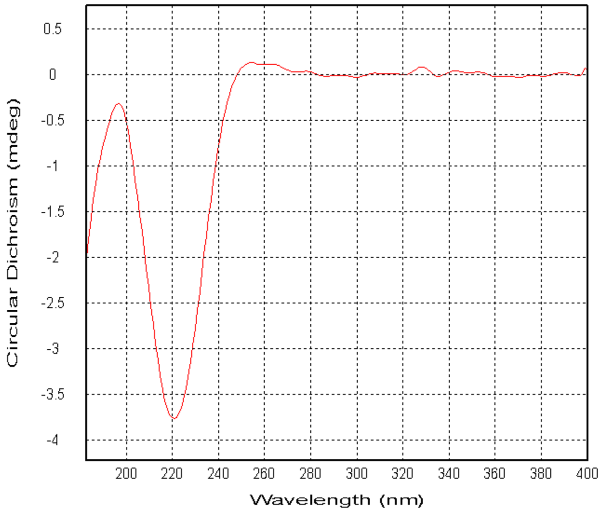

4

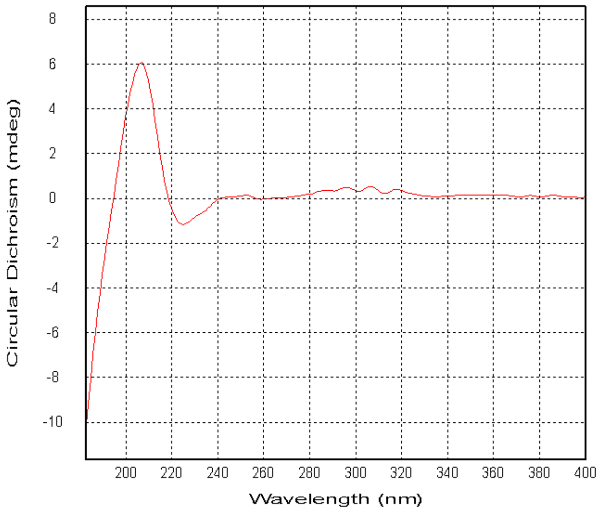

5

Figure S2. The CD spectra of compounds **3**–**5** in ACN.

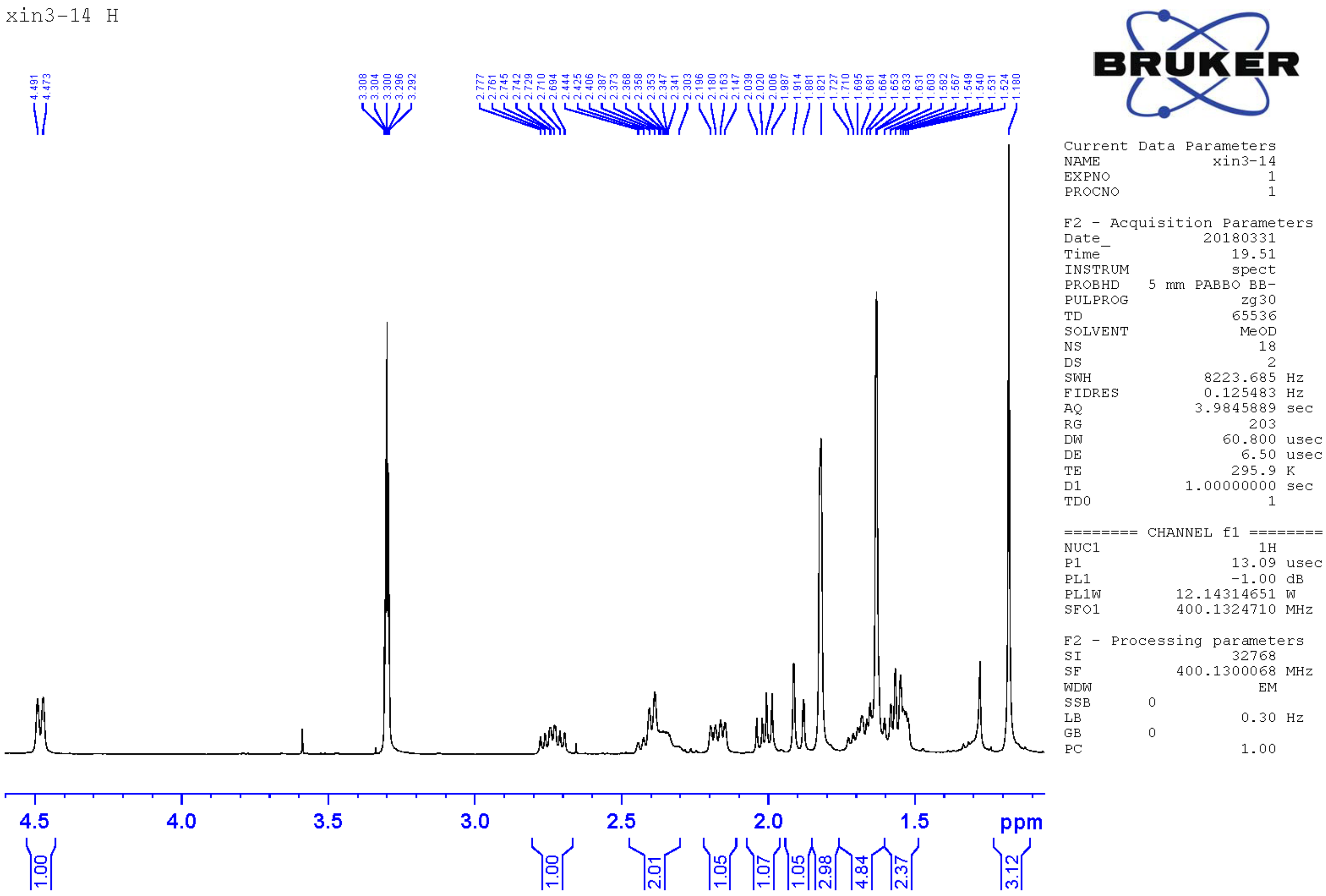

Figure S3. ^{1}H NMR spectrum of **1** in CD_3OD.

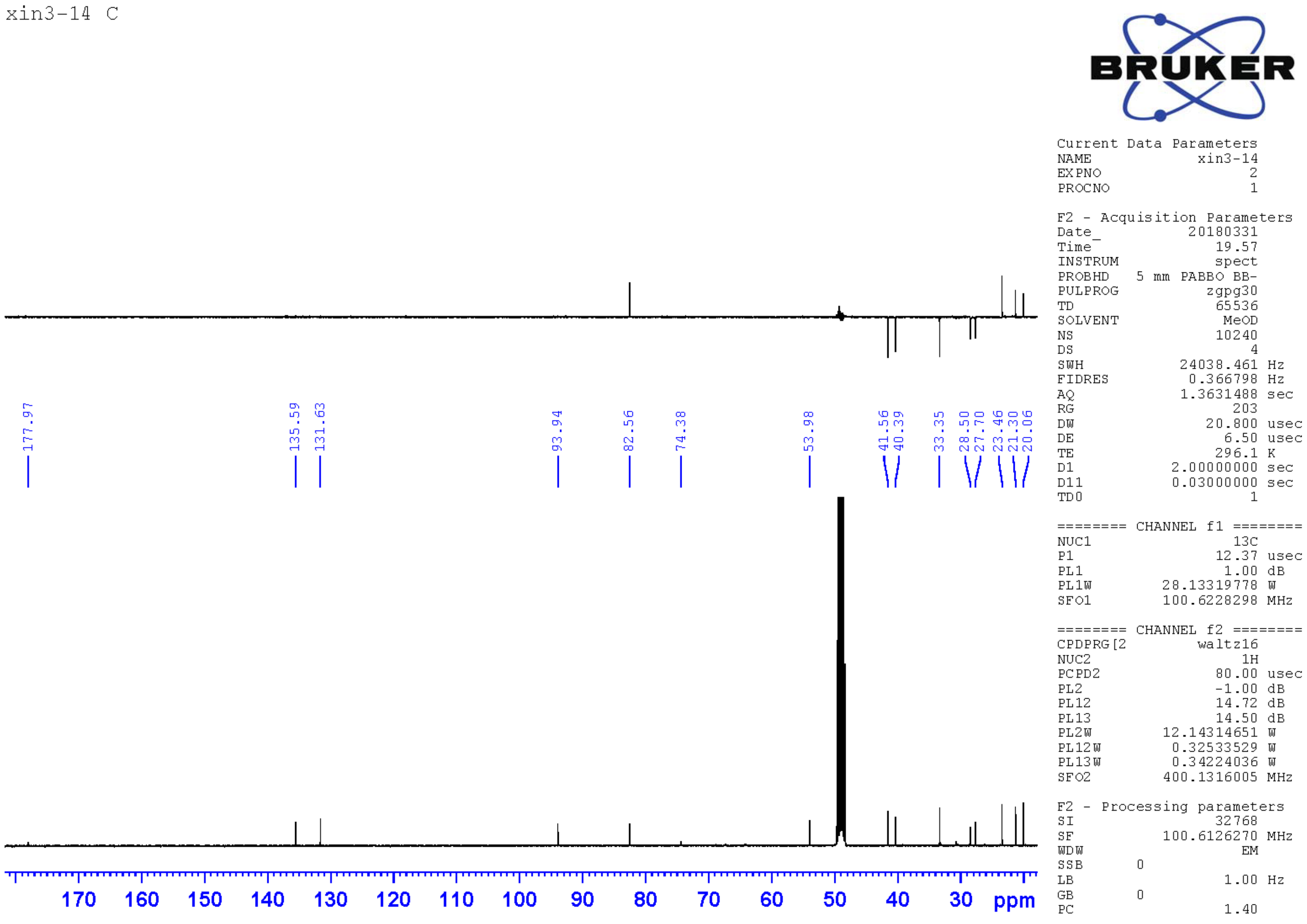

Figure S4. ^{13}C NMR spectrum of **1** in CD_3OD.

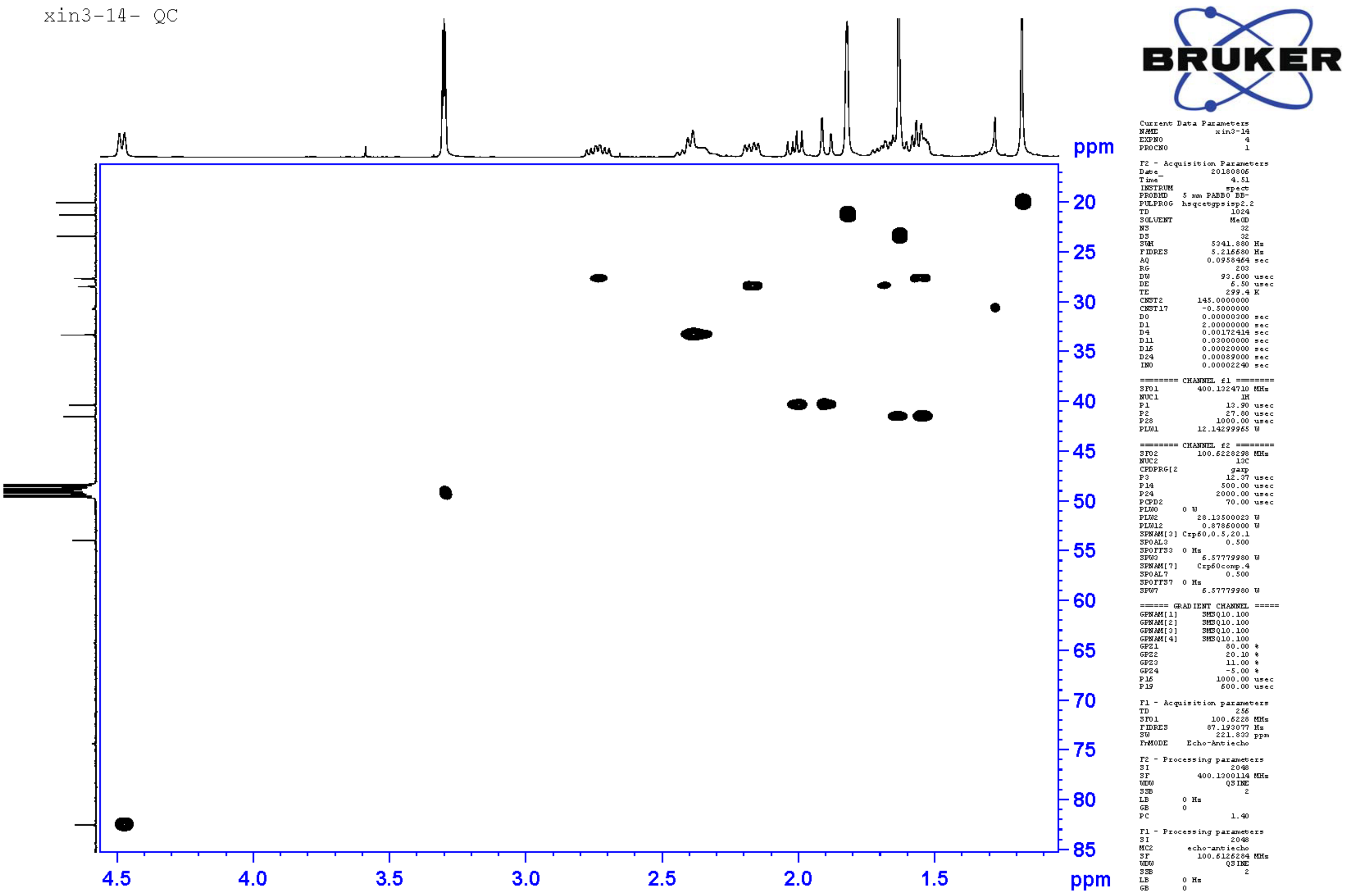

Figure S5. HSQC spectrum of **1** in CD_3OD.

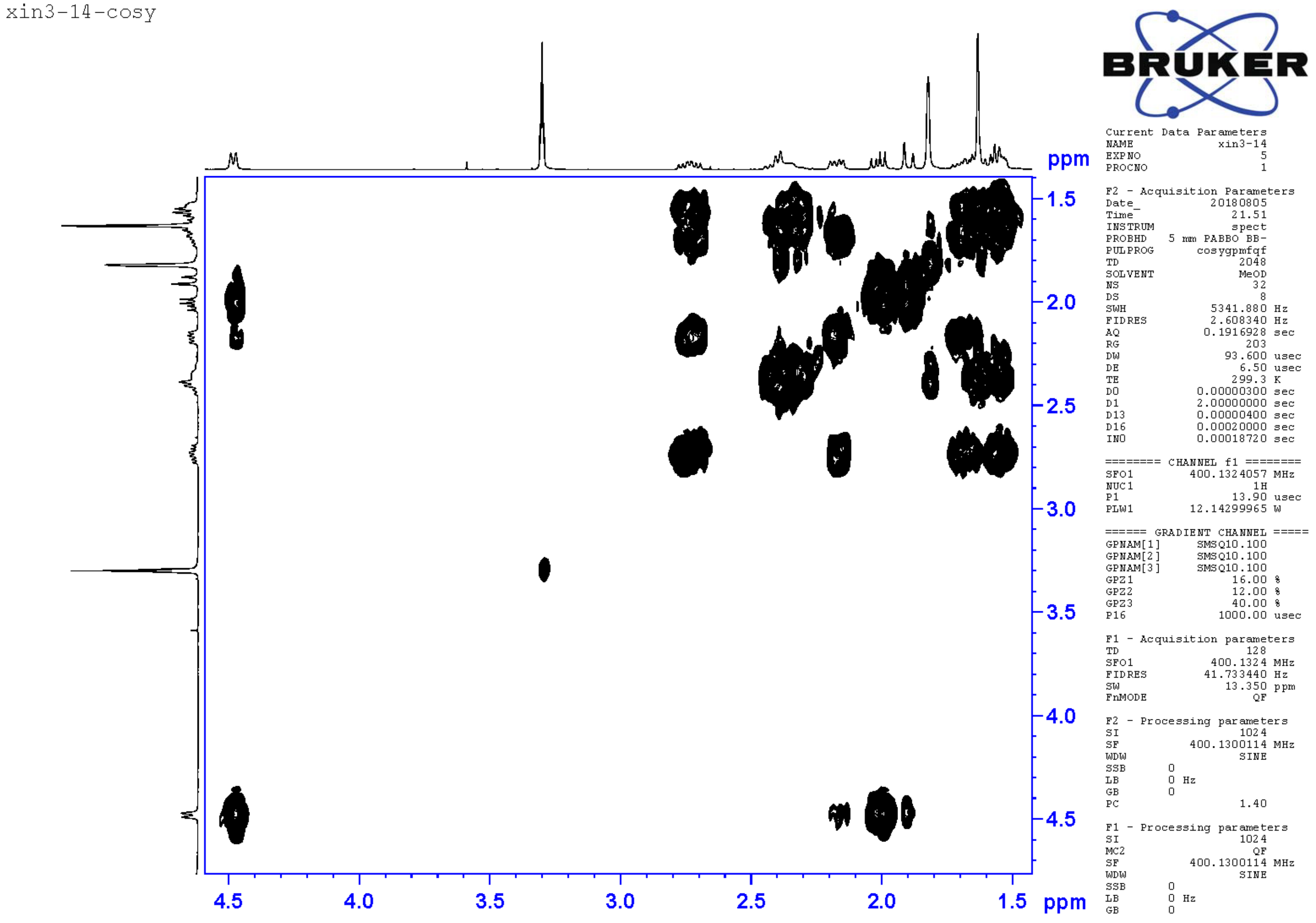

Figure S6. COSY NMR spectrum of **1** in CD_3OD.

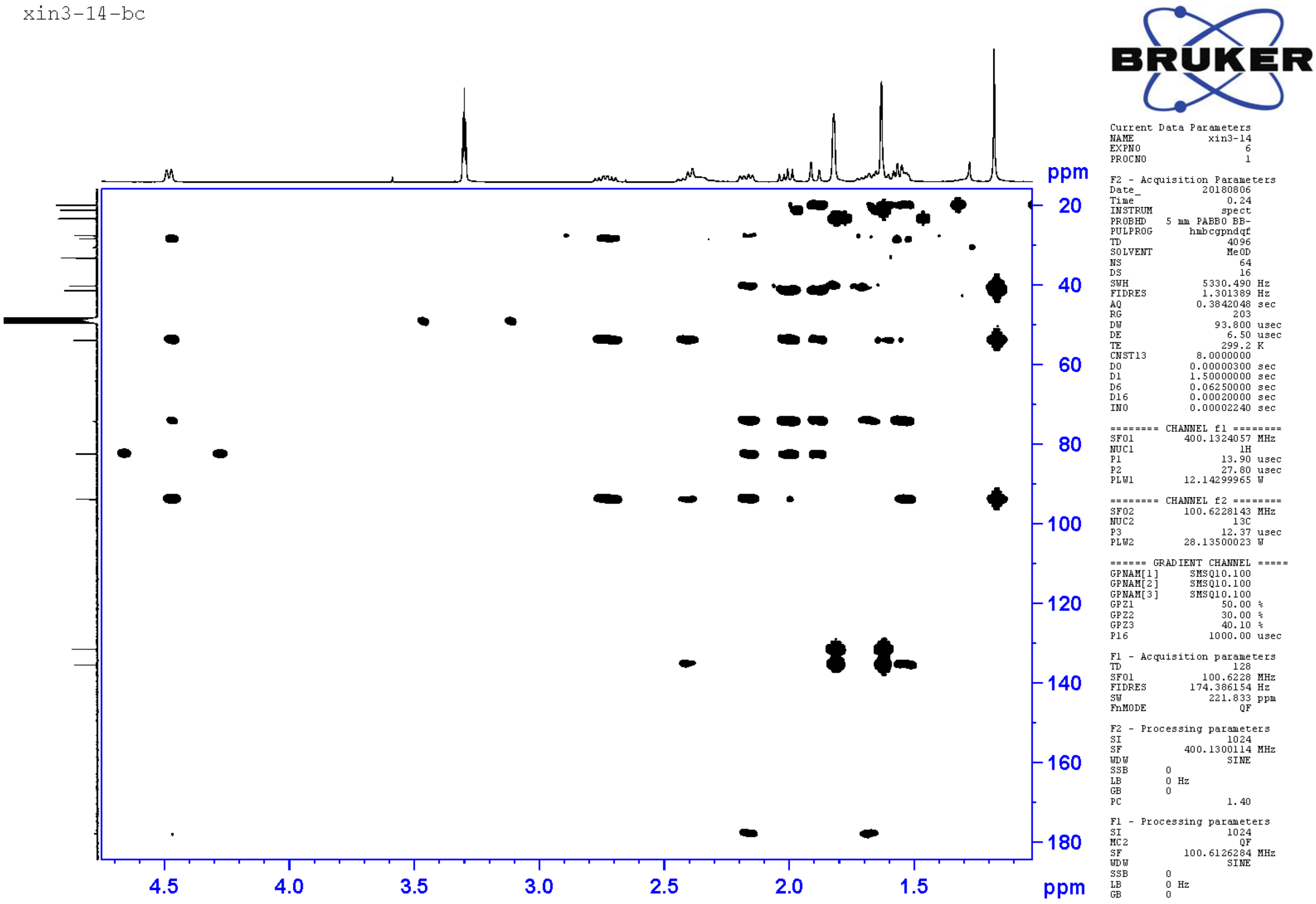

Figure S7. HMBC NMR spectrum of **1** in CD_3OD.

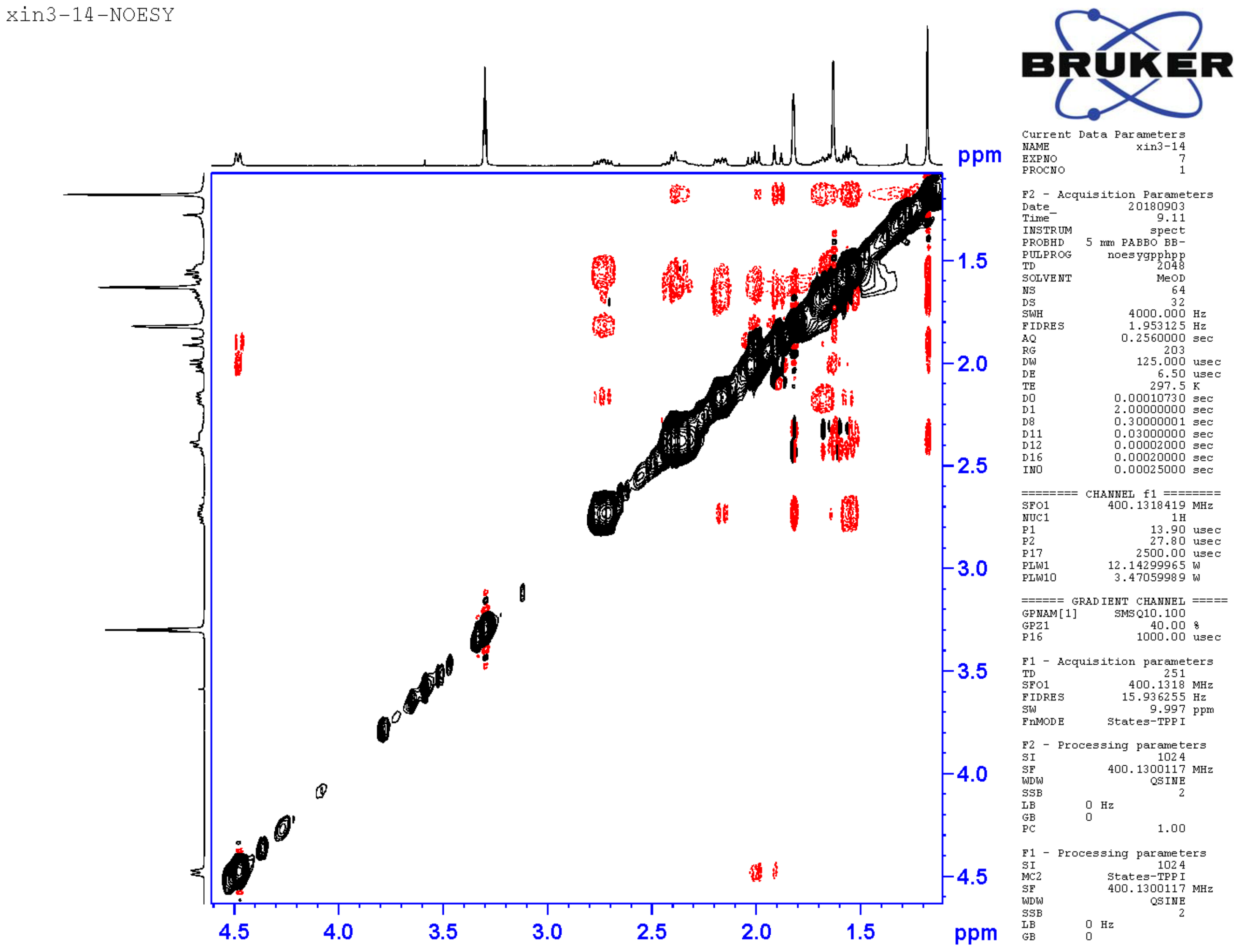

Figure S8. NOESY NMR spectrum of **1** in CD_3OD.

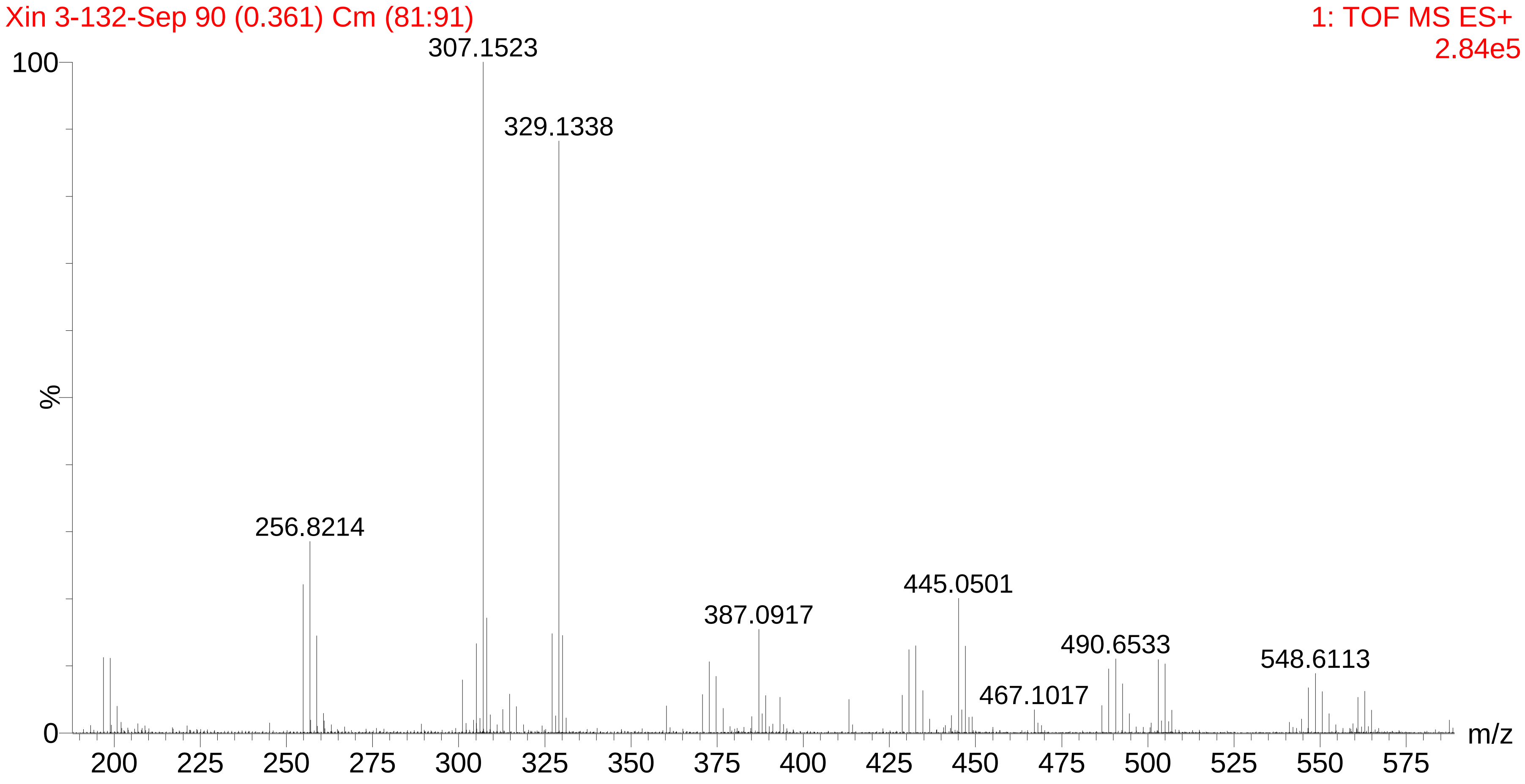

Figure S9. The HR-ESI-MS spectrum of **1**.

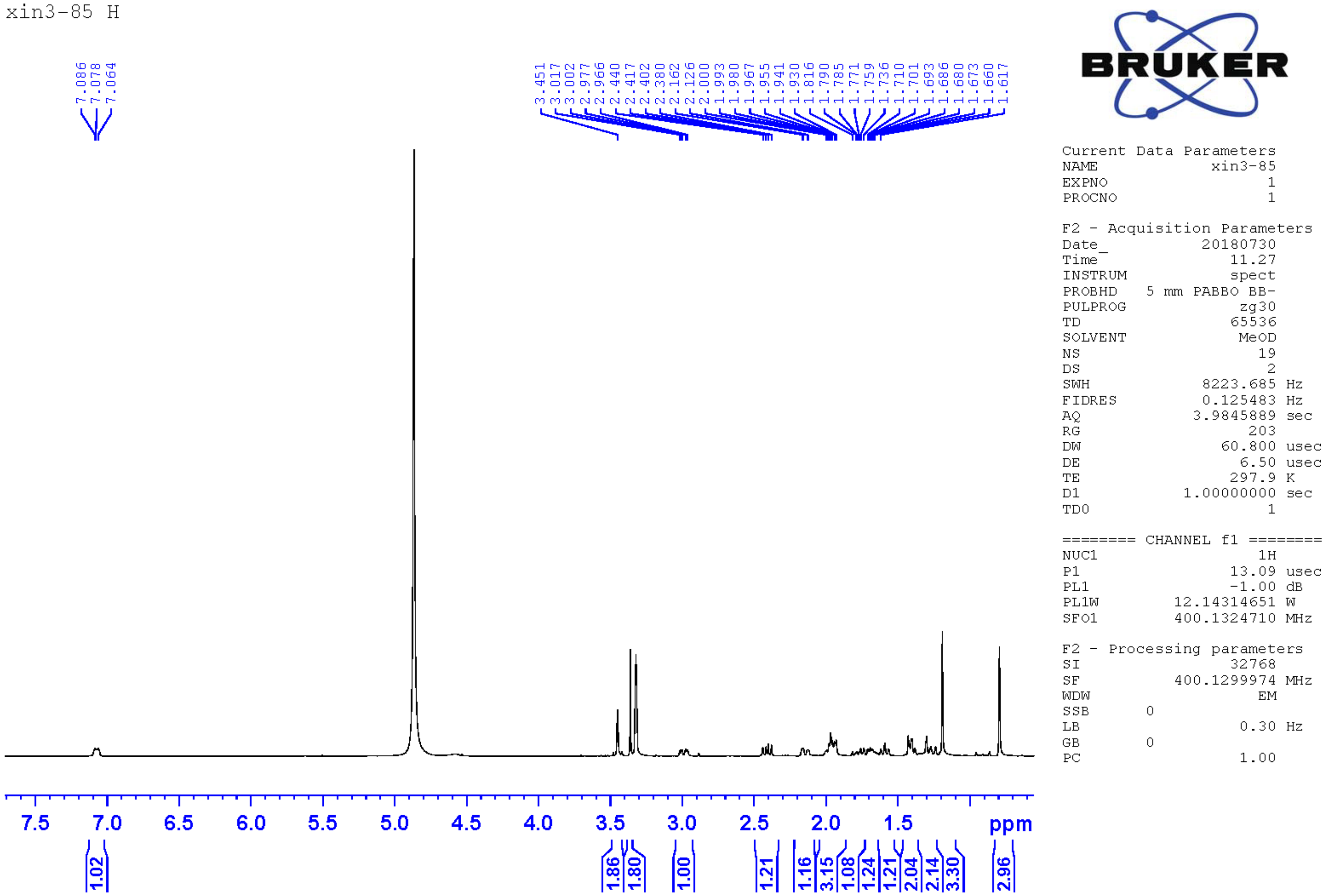

Figure S10. ^{1}H NMR spectrum of **2** in CD_3OD.

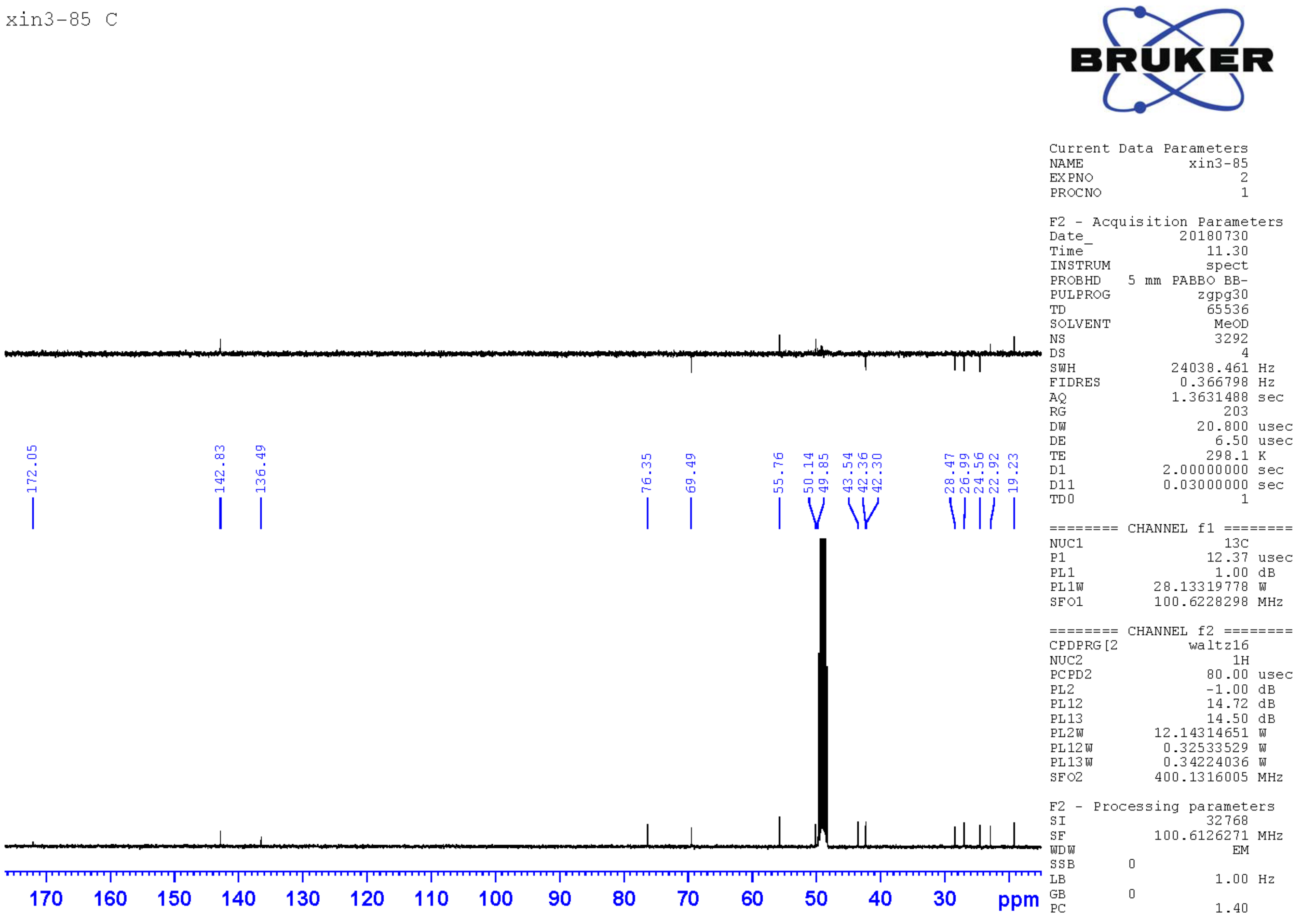

Figure S11. ^{13}C NMR spectrum of **2** in CD_3OD.

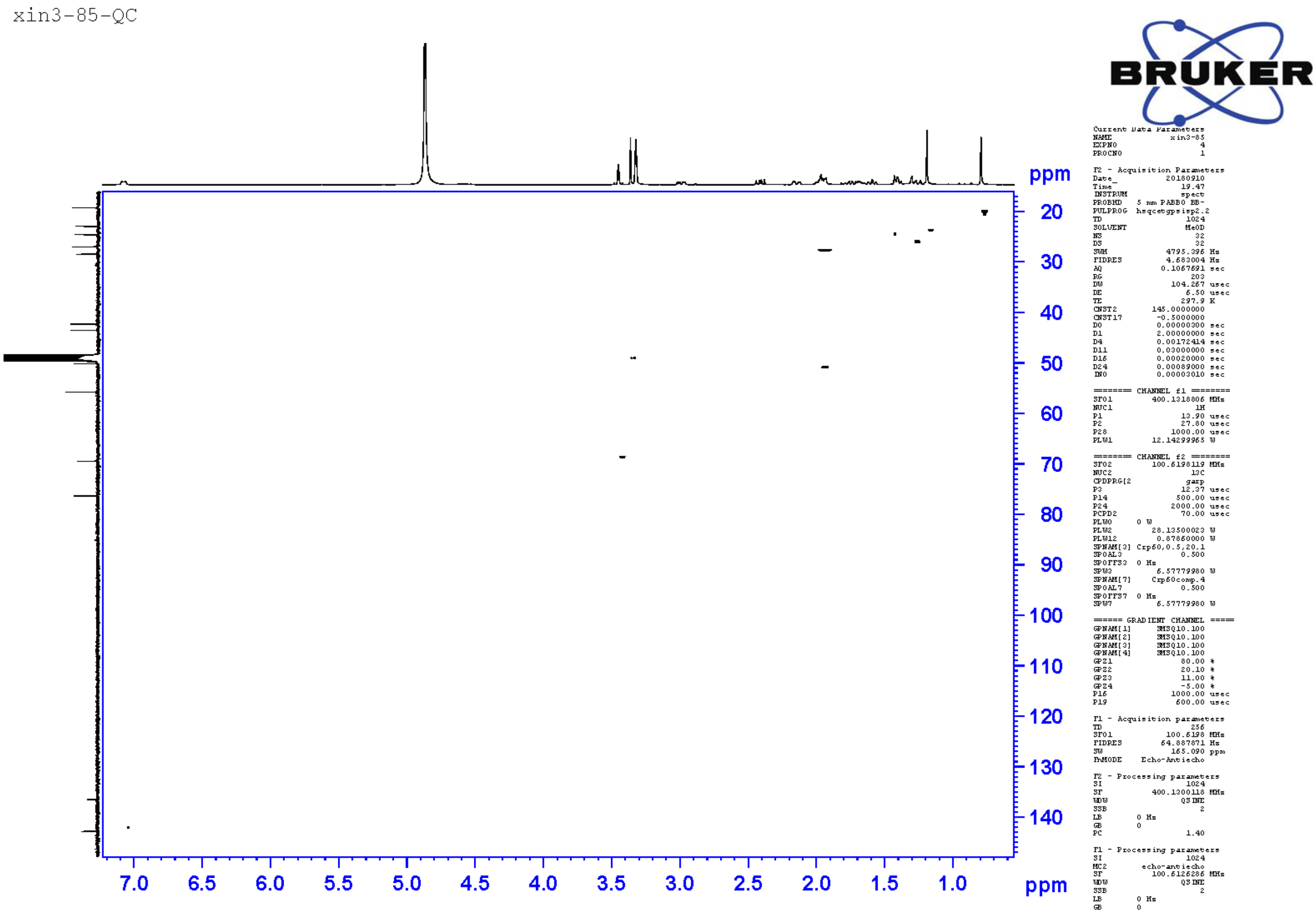

Figure S12. HSQC spectrum of **2** in CD_3OD.

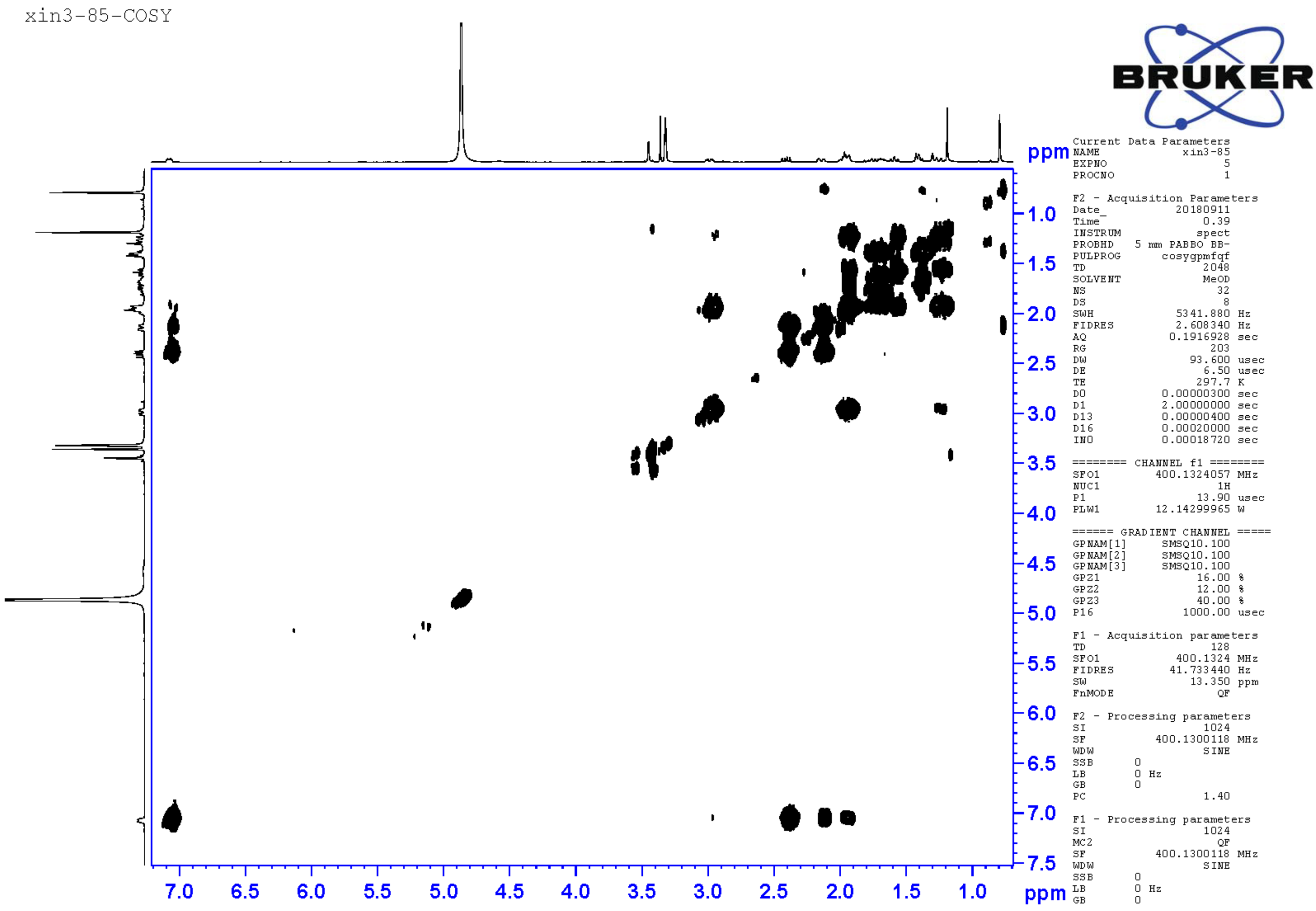

Figure S13. COSY NMR spectrum of **2** in CD_3OD.

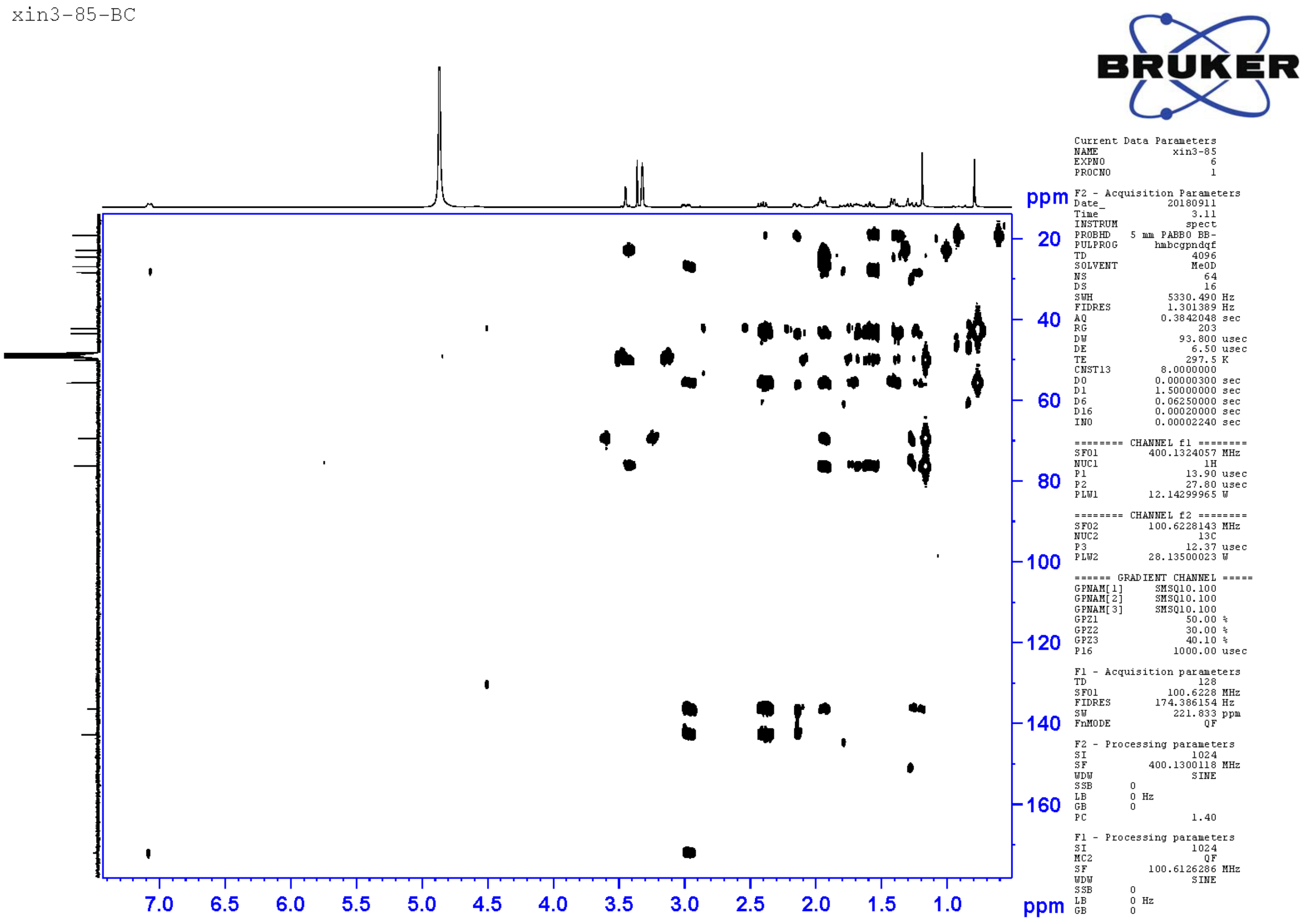

Figure S14. HMBC NMR spectrum of **2** in CD_3OD.

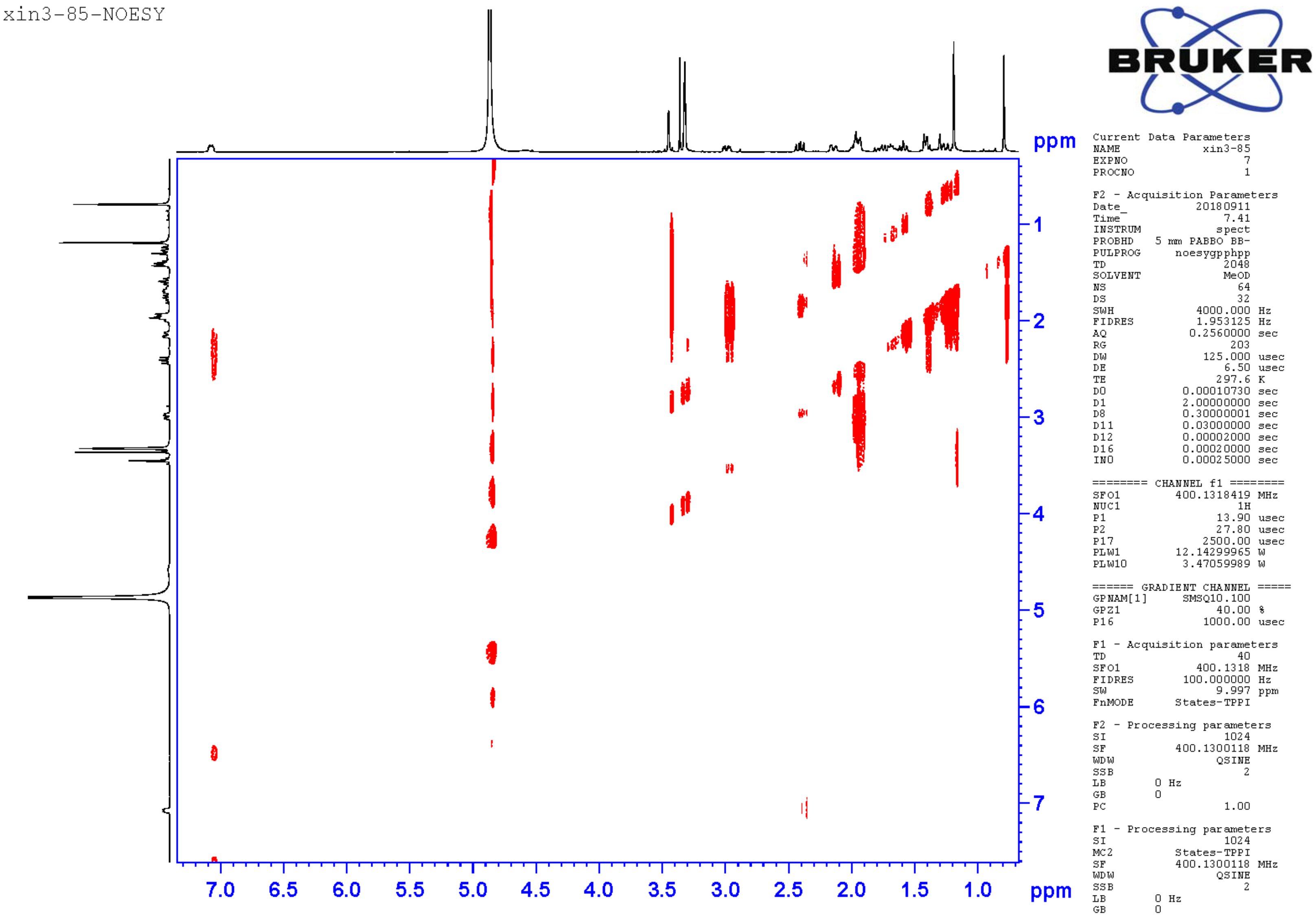

Figure S15. NOESY NMR spectrum of **2** in CD_3OD.

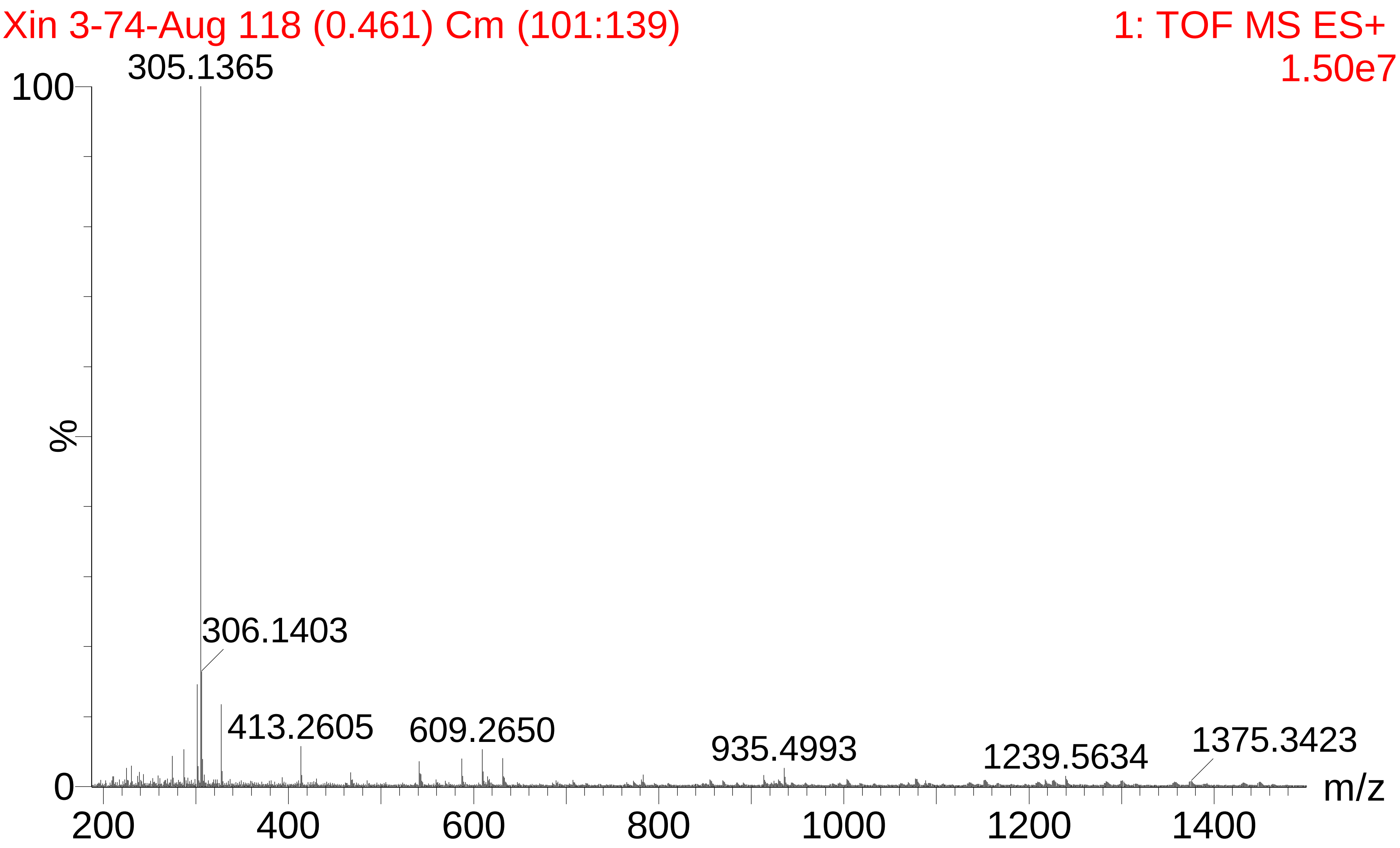

Figure S16. The HR-ESI-MS spectrum of **2**.

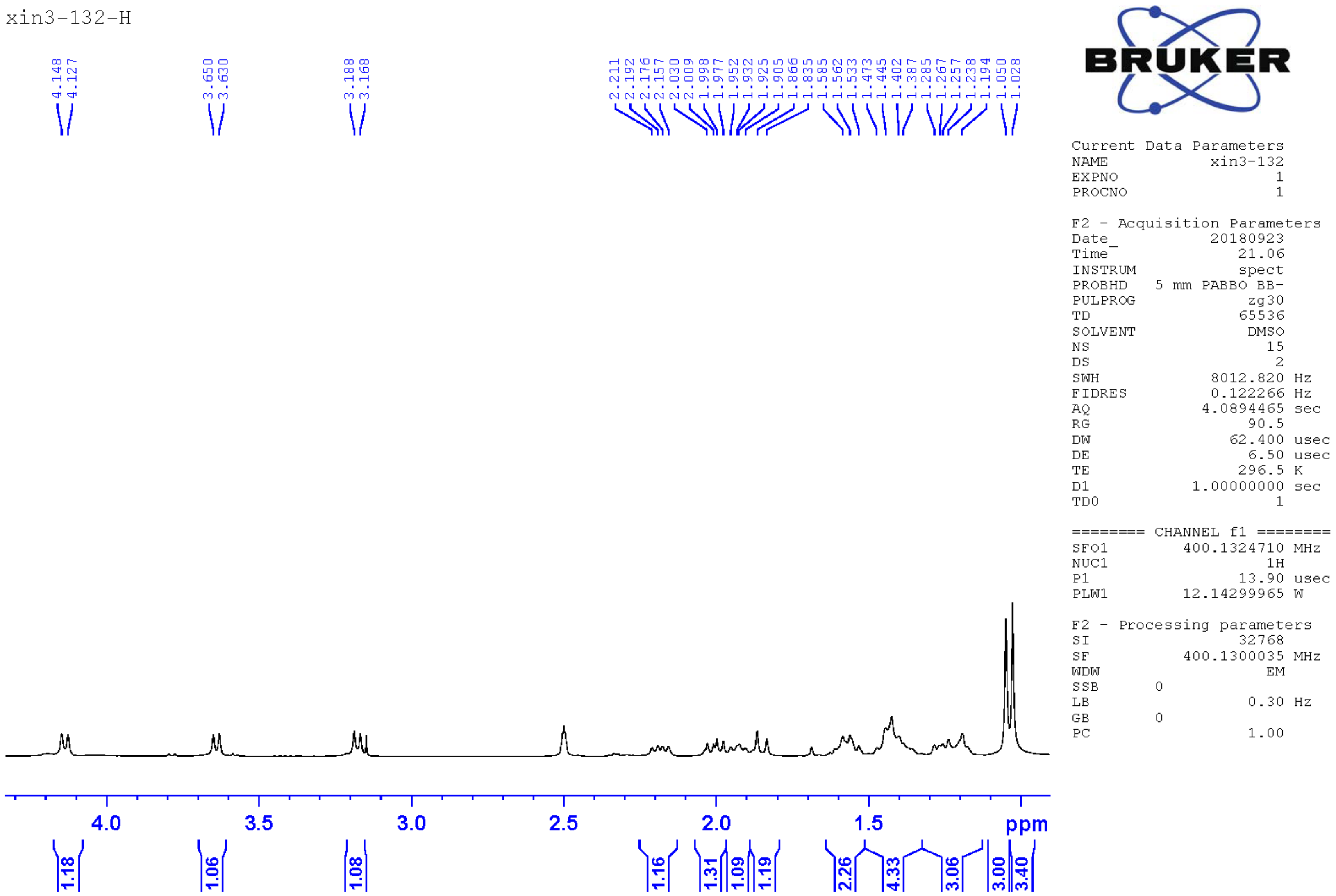

Figure S17. ^{1}H NMR spectrum of **3** in DMSO-d_6.

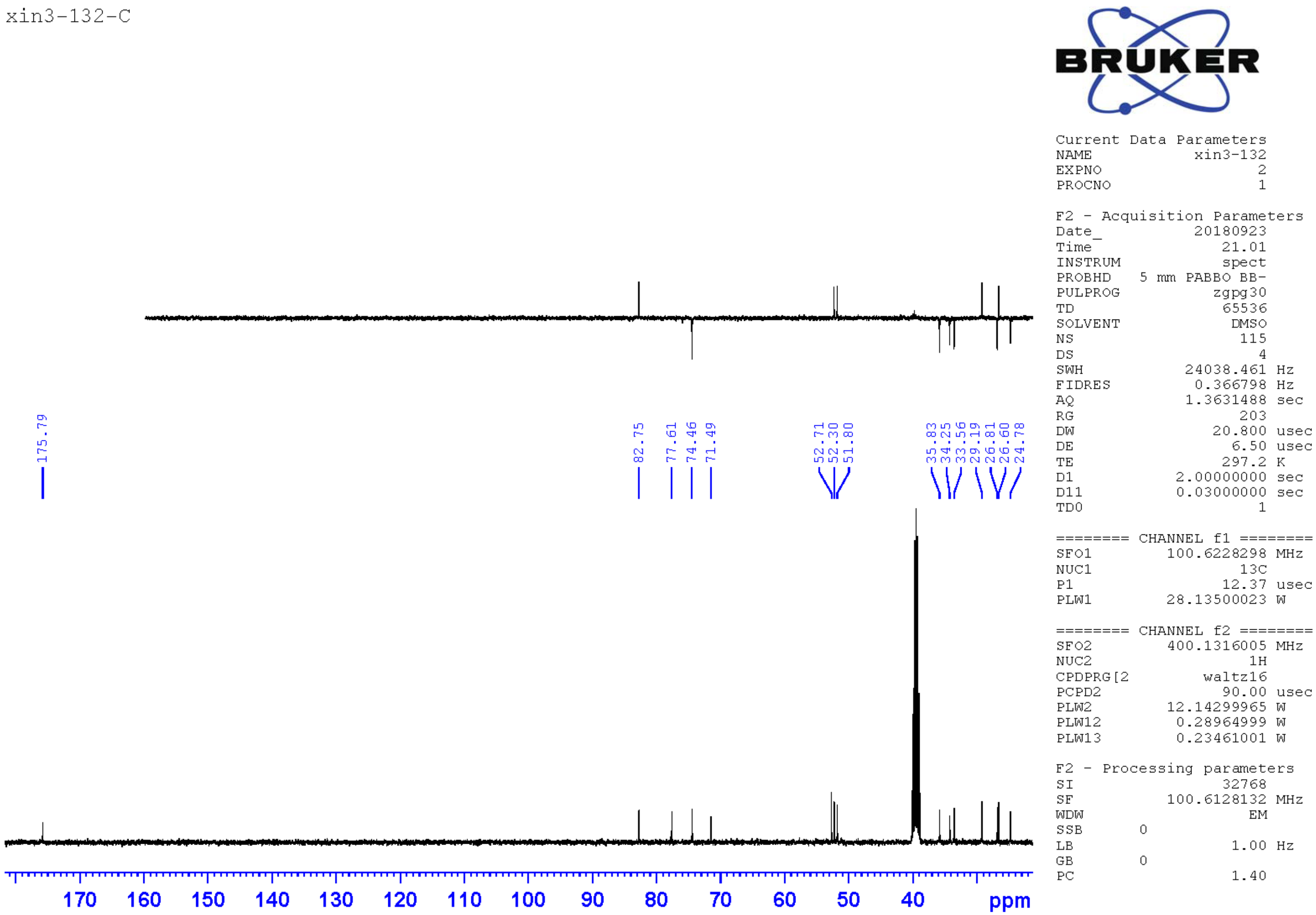

Figure S18. ^{13}C NMR spectrum of **3** in DMSO-d_6.

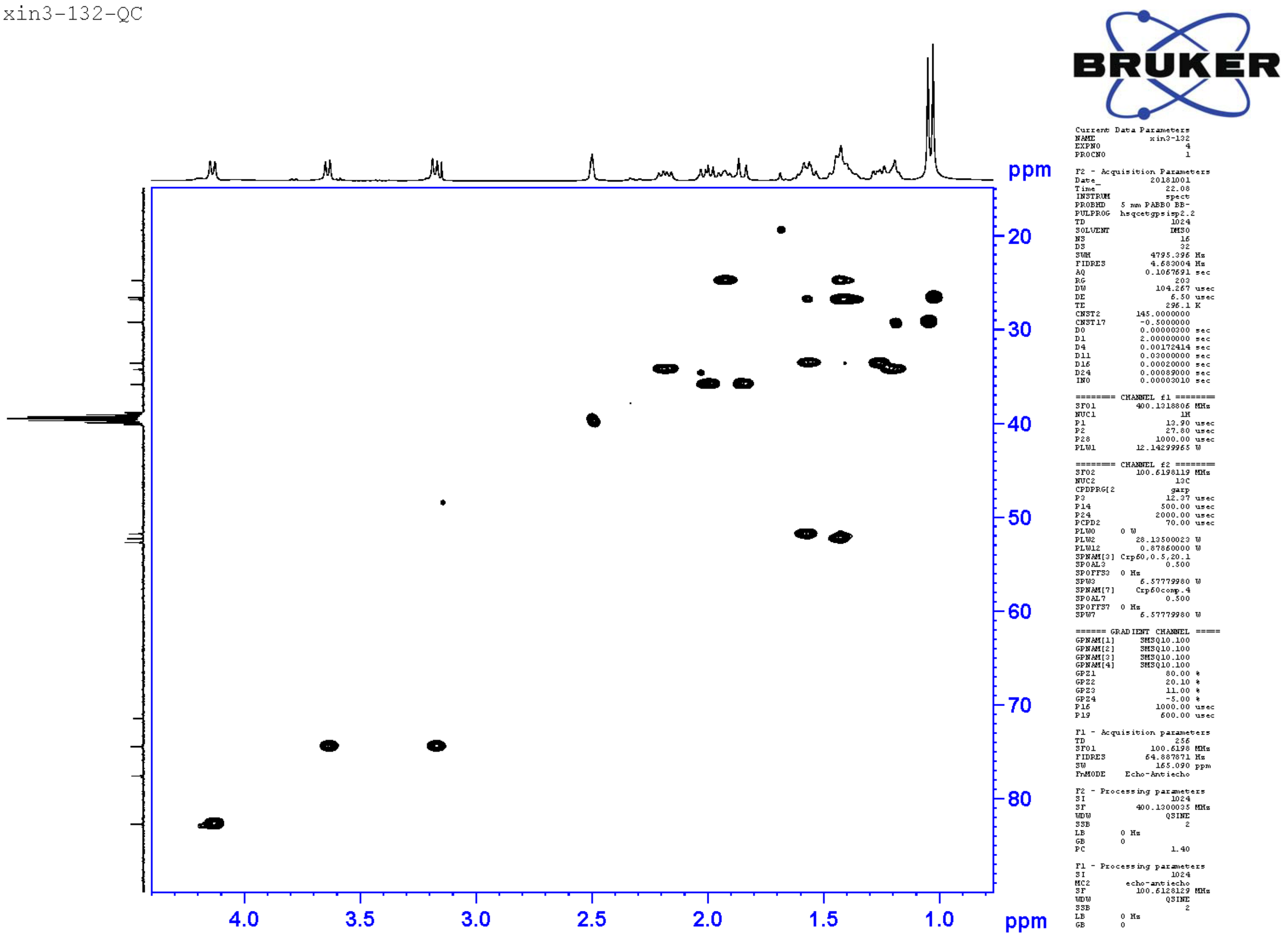

Figure S19. HSQC spectrum of **3** in DMSO-d_6.

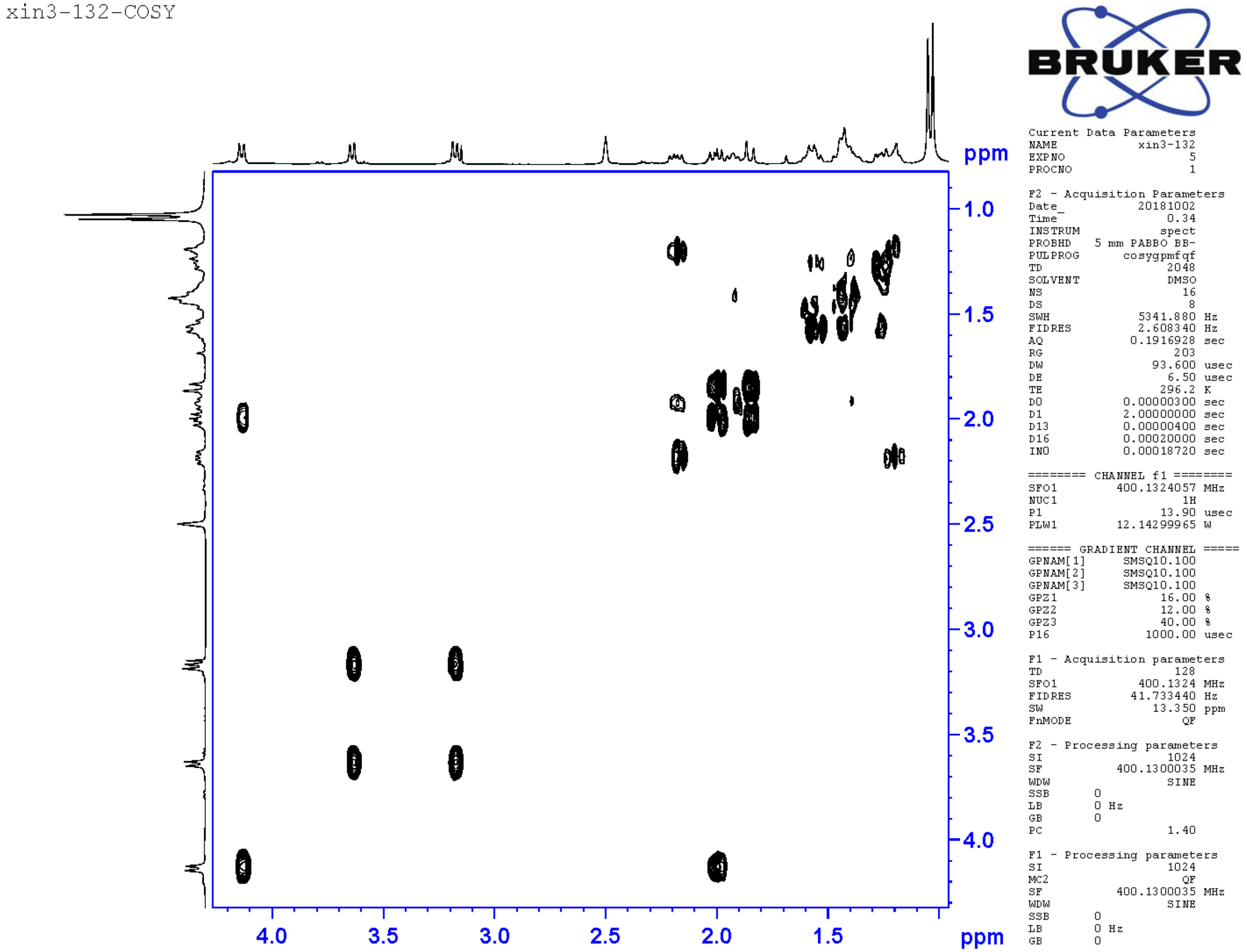

Figure S20. COSY NMR spectrum of **3** in DMSO-d_6.

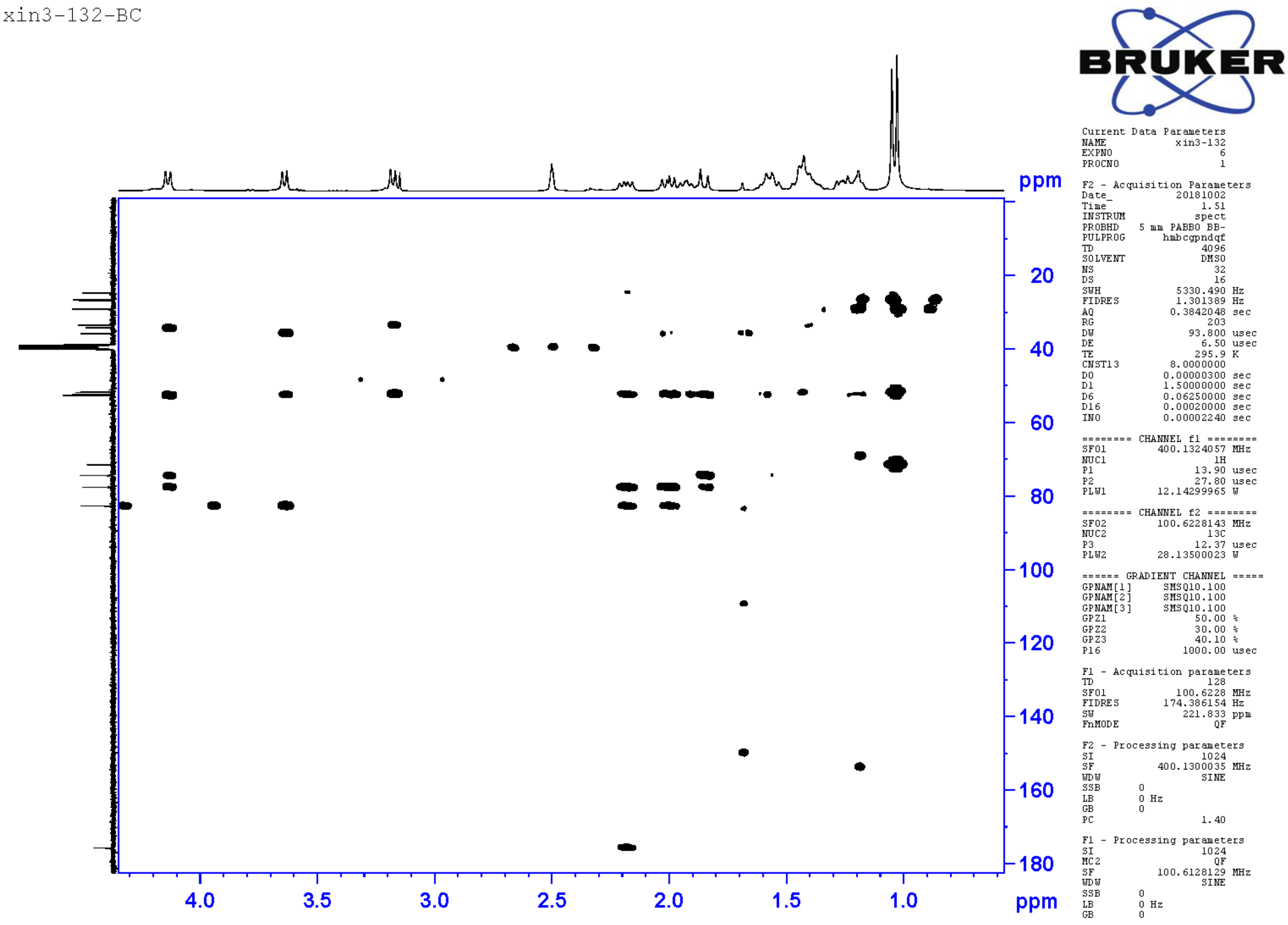

Figure S21. HMBC NMR spectrum of **3** in DMSO-d_6.

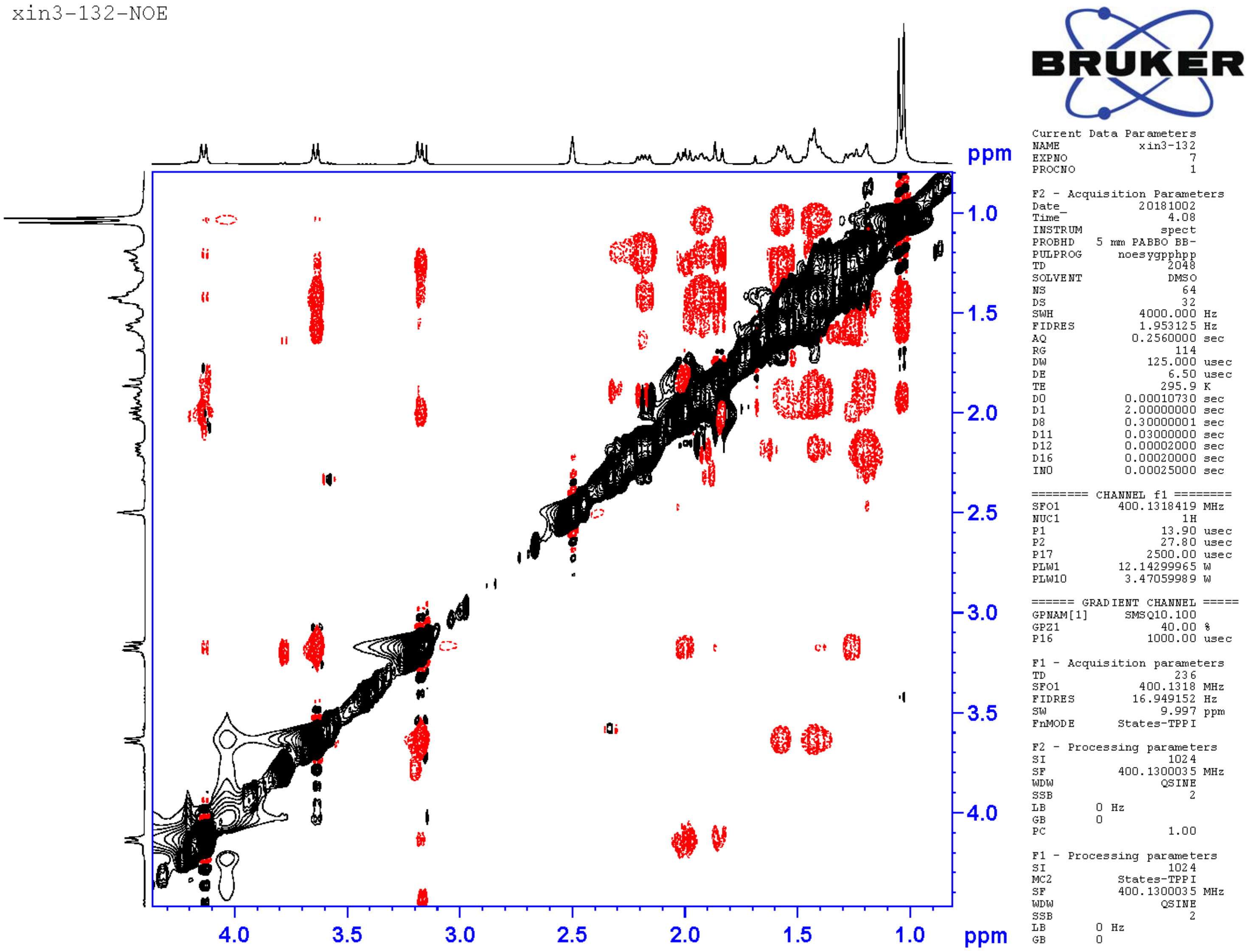

Figure S22. NOESY NMR spectrum of **3** in DMSO-d_6.

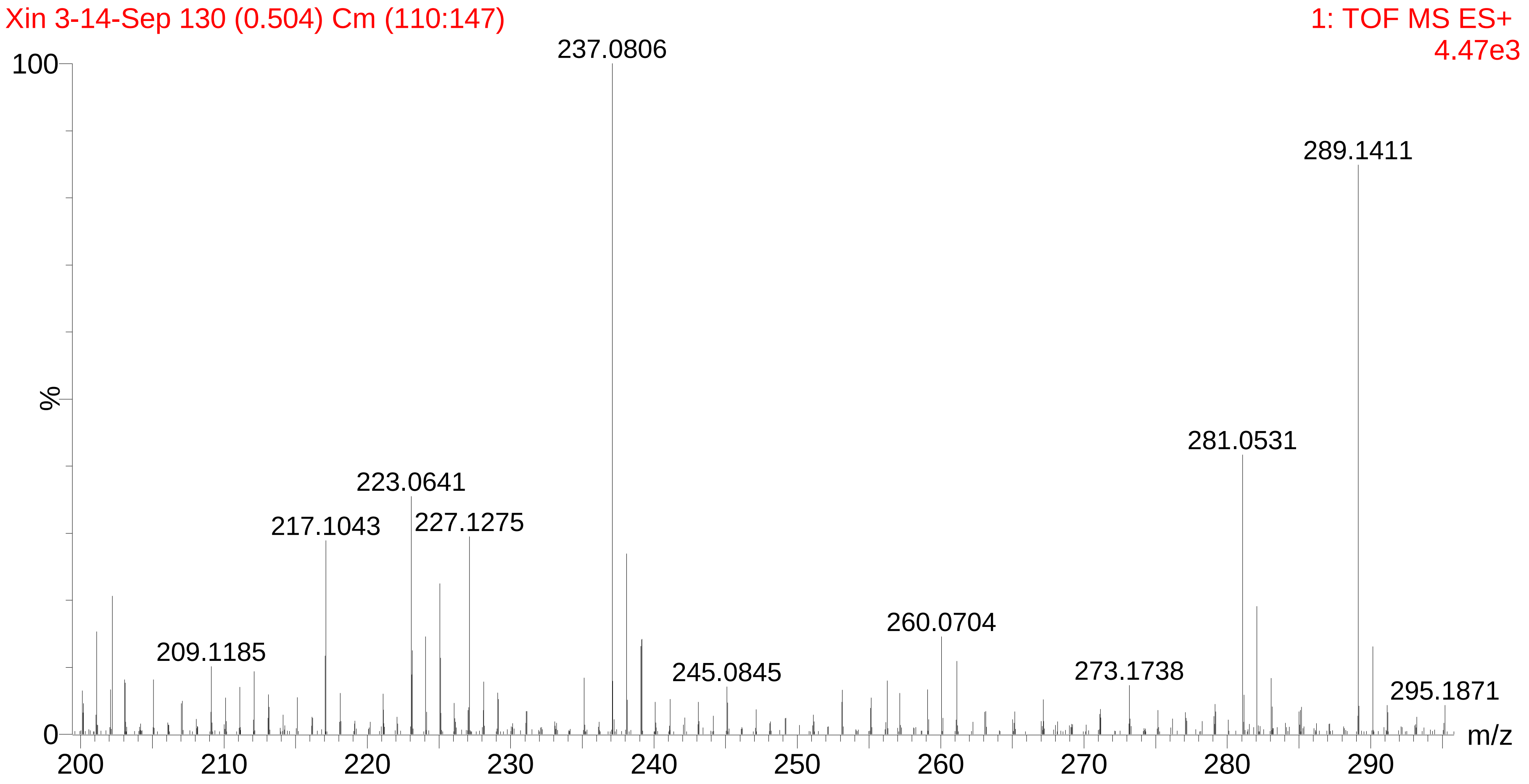

Figure S23. The HR-ESI-MS spectrum of **3**.

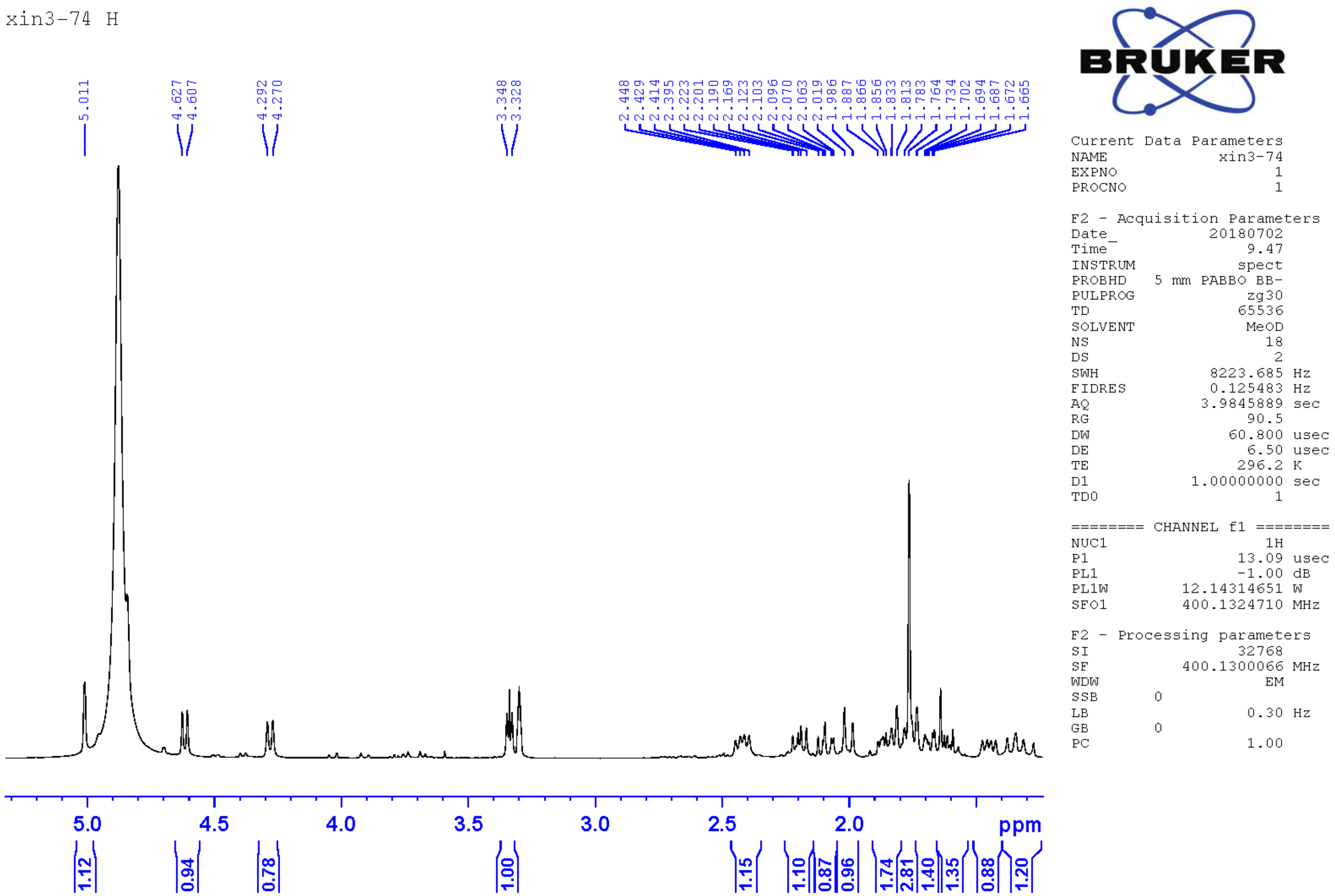

Figure S24. ^{1}H NMR spectrum of **4** in CD_3OD.

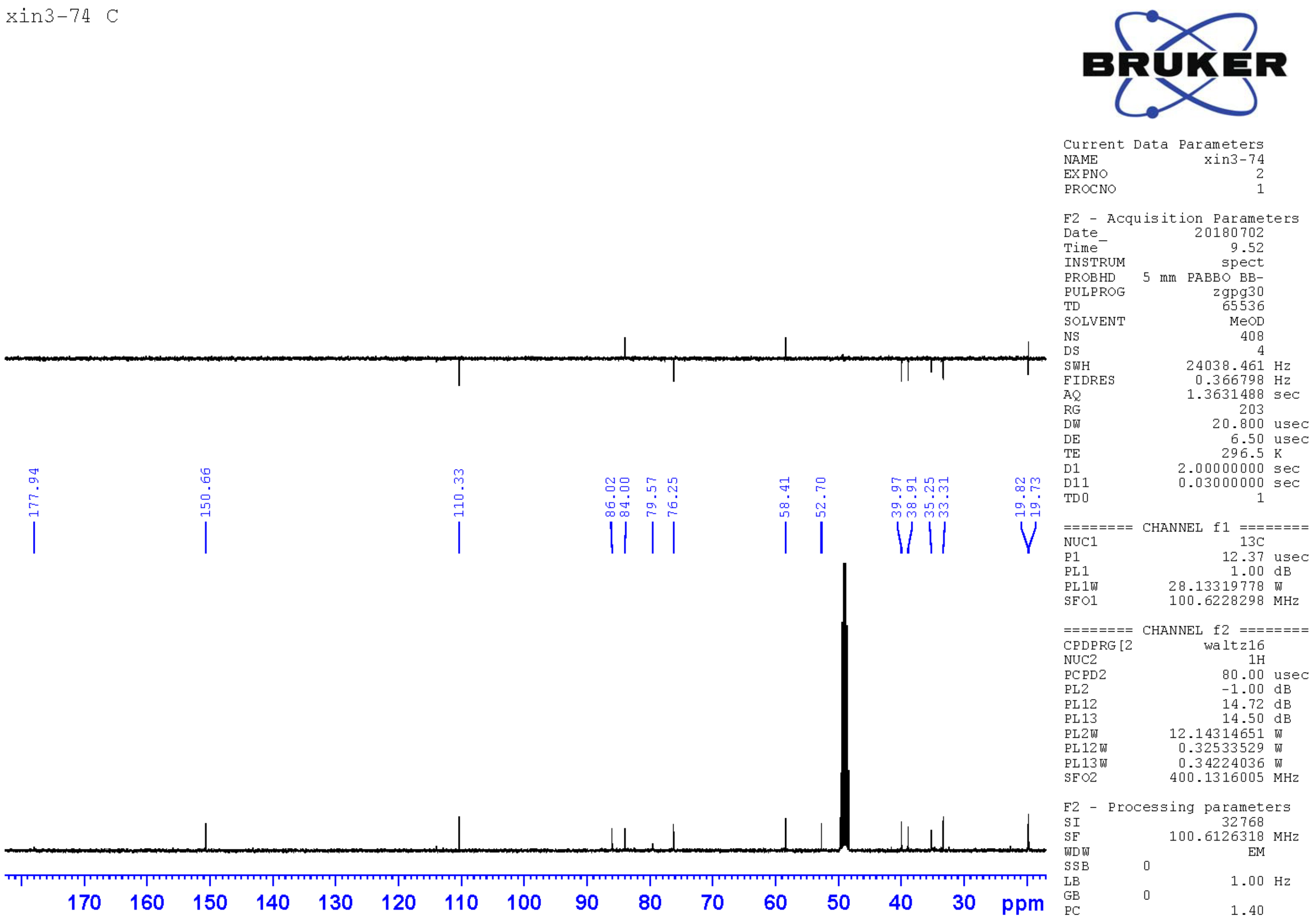

Figure S25. ^{13}C NMR spectrum of **4** in CD_3OD.

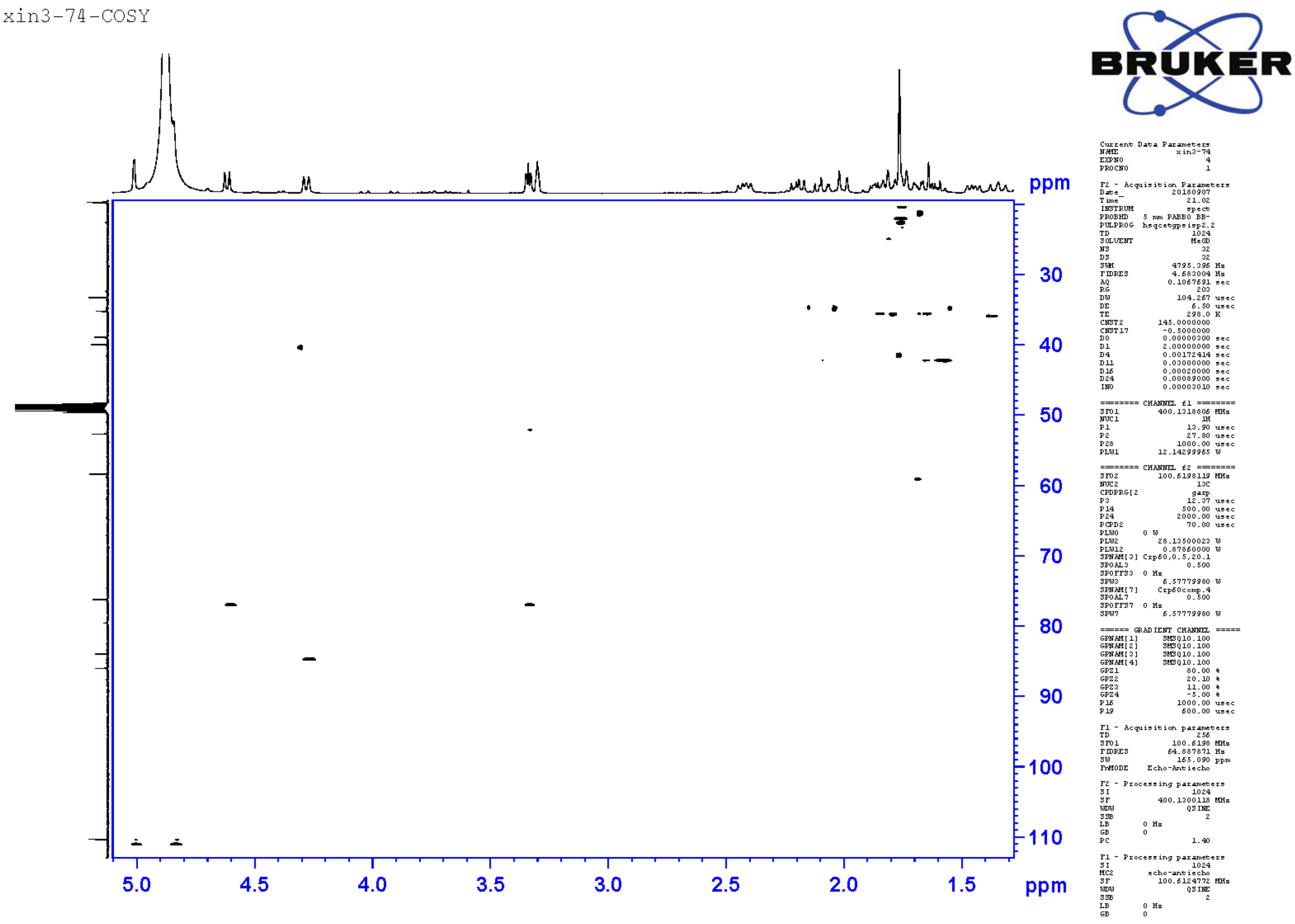

Figure S26. HSQC spectrum of **4** in CD_3OD.

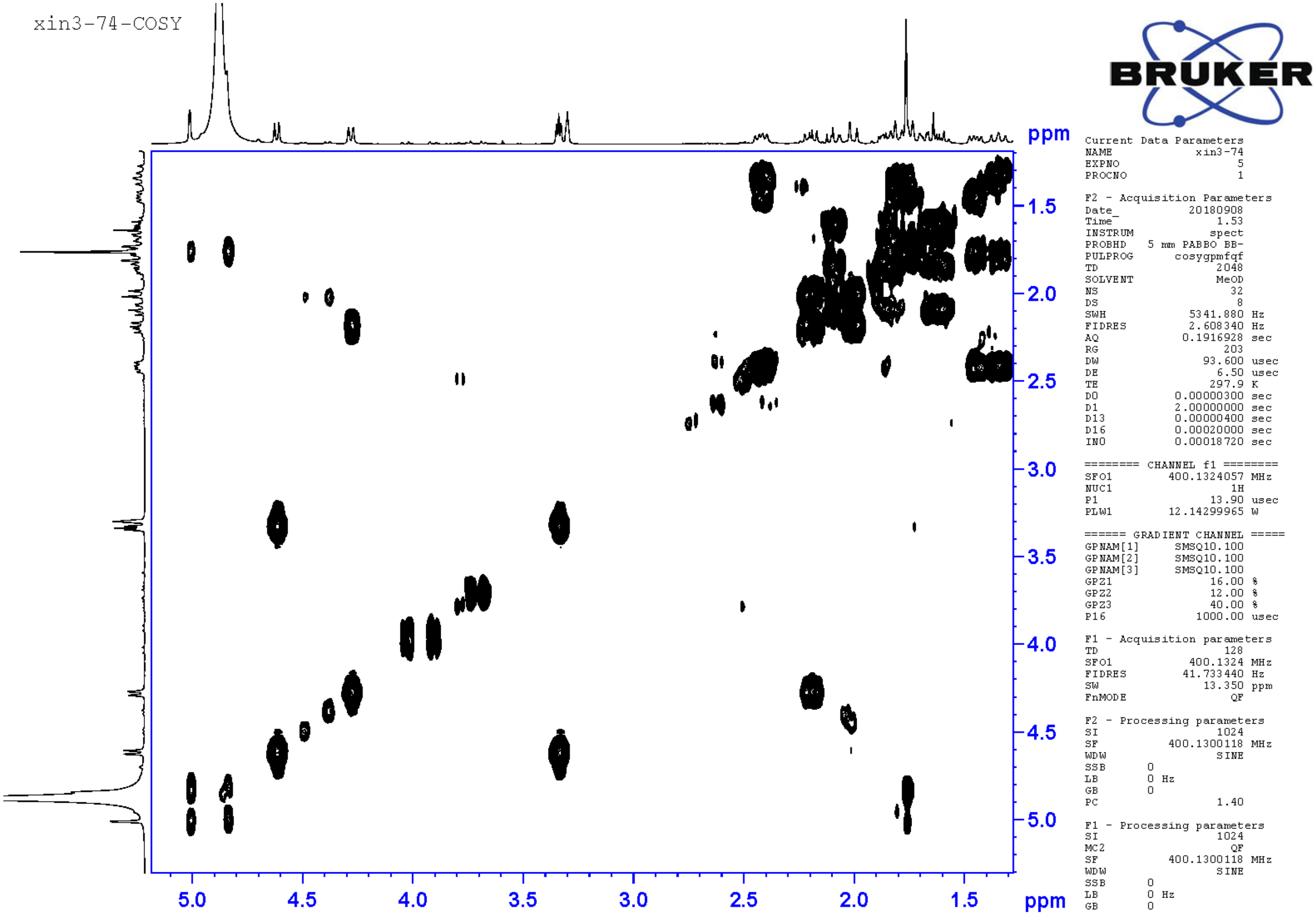

Figure S27. COSY NMR spectrum of **4** in CD_3OD.

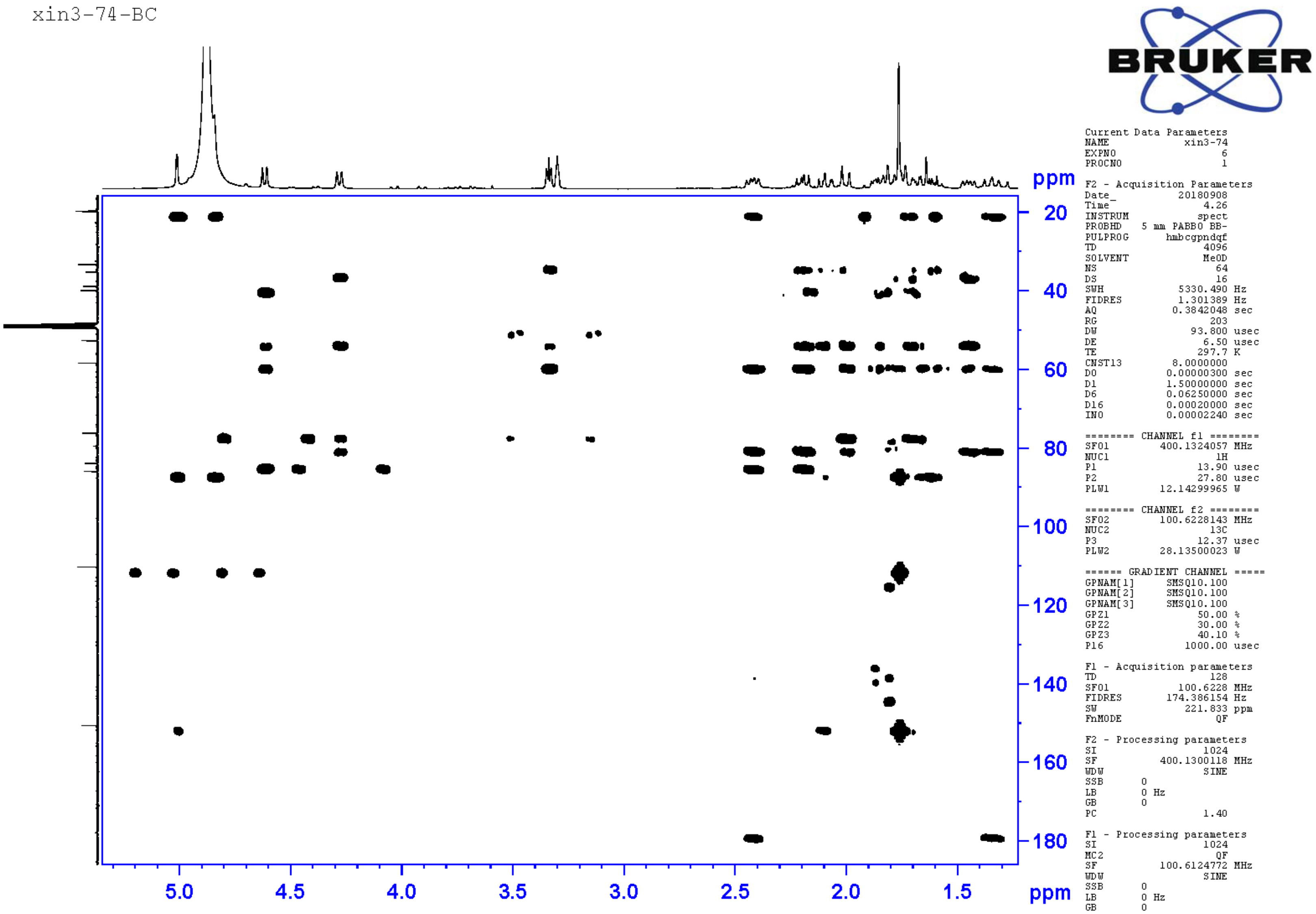

Figure S28. HMBC NMR spectrum of **4** in CD_3OD.

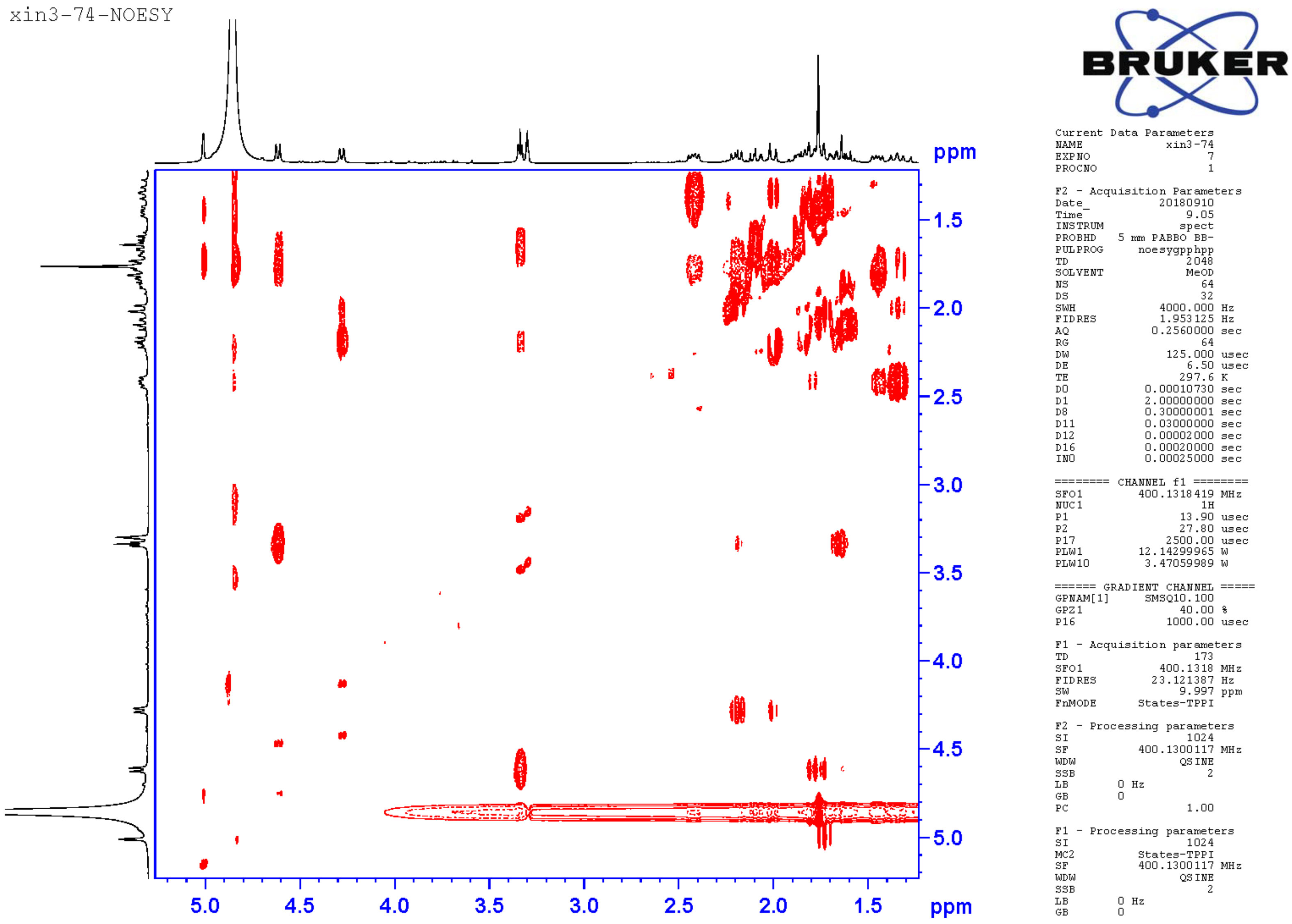

Figure S29. NOESY NMR spectrum of **4** in CD_3OD.

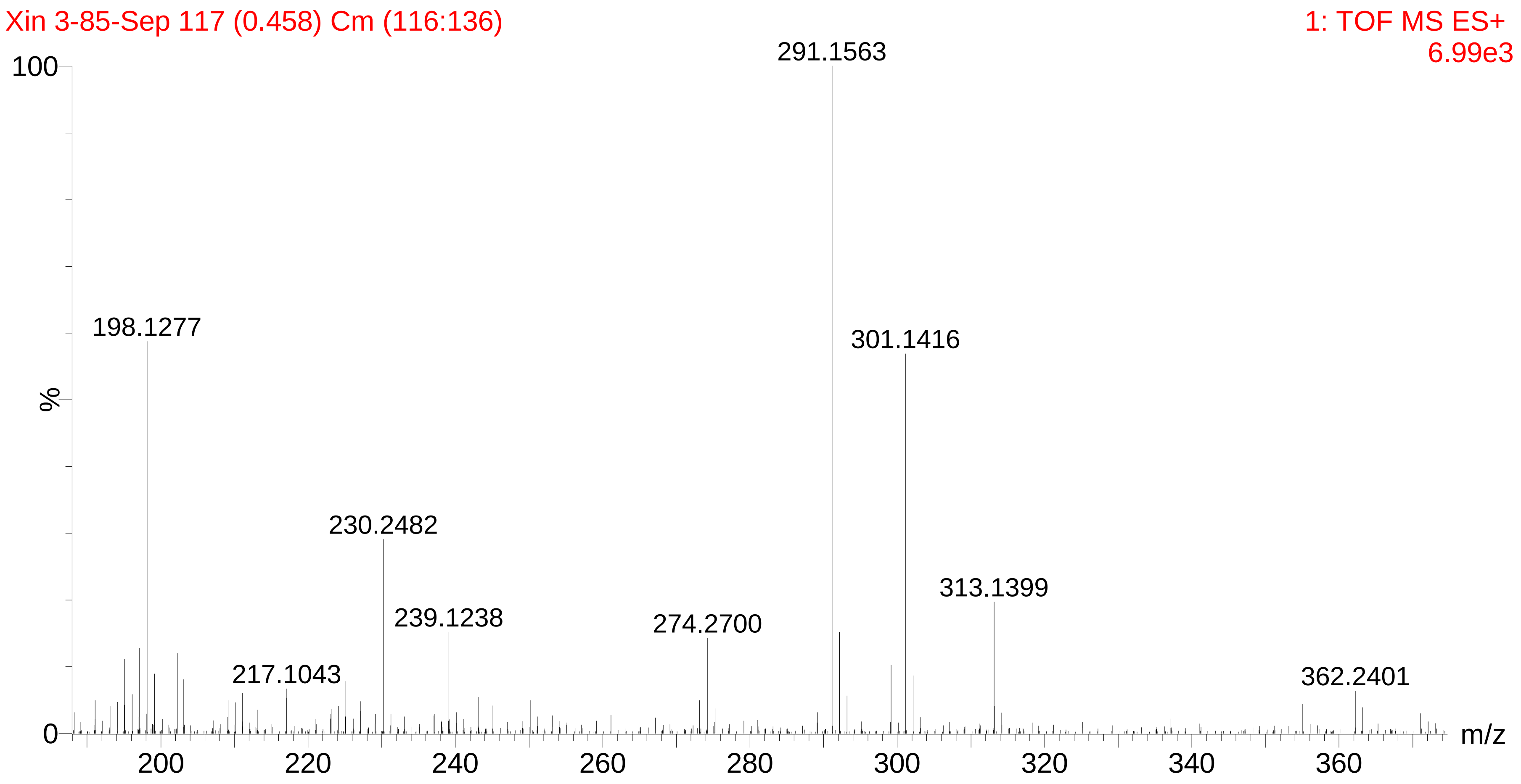

Figure S30. The HR-ESI-MS spectrum of **4**.
